# Depth-Dependent Emission from Silver Dopants in Single CdSe Nanoplatelets

**DOI:** 10.1021/acsnano.5c11745

**Published:** 2026-02-04

**Authors:** Mitesh Amin, Farwa Awan, Michael W. Swift, William Girten, Sean W. O’Neill, Steven C. Erwin, Alexander L. Efros, Todd D. Krauss

**Affiliations:** † The Institute of Optics, 6927 University of Rochester, Rochester, New York 14627, United States; ‡ Department of Chemistry, University of Rochester, Rochester, New York 14627, United States; § Center for Computational Materials Science, 41487 U.S. Naval Research Laboratory, Washington D.C. 20375, United States; ∥ University of Rochester Integrated Nanosystems Center (URnano), University of Rochester, Rochester, New York 14627, United States

**Keywords:** nanoplatelets, colloidal nanocrystals, dopants, photoluminescence, single photon sources, single particle spectroscopy

## Abstract

Dopants in semiconductor nanostructures offer tremendous control over electronic, optical, and magnetic properties beyond what is achievable in bulk materials. We demonstrate that the broad dopant emission in semiconductor nanoplatelets effectively maps the electron wave function across the nanoplatelet thickness. Both the emission energy and lifetime of the dopant transition depend strongly on the depth of the dopant within the nanoplatelet. This dependence arises from the electrostatic self-interaction of the charged dopant, which varies with proximity to the dielectric discontinuity at the nanoplatelet surface. Through comprehensive single-particle spectroscopy of silver-doped CdSe nanoplatelets, we verify that acceptors near the center emit at higher energies with shorter lifetimes, while those near the surface emit at lower energies with longer lifetimes. This spatial mapping also reveals unusual two-color emission from individual nanoplatelets, with enhanced Auger recombination yielding exceptional photon antibunching (>90% purity) at room temperature, suggesting potential applications in quantum information technologies.

## Graphical abstract

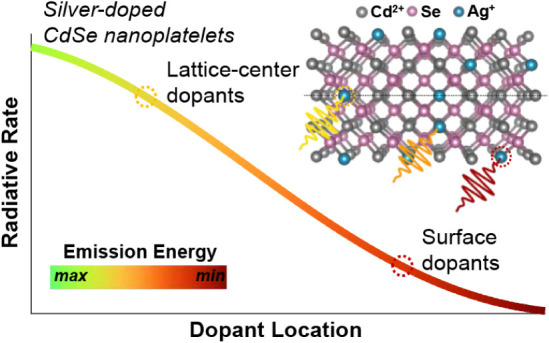

Incorporating dopants into colloidal semiconductor nanocrystals (NCs) provides an opportunity to engineer truly novel materials with precise control over optoelectronic properties, by leveraging the complex interplay between dopants, external environments, and quantum confinement.^1,2^ Dopant incorporation occurs via cation-exchange chemistry, which remarkably preserves NC morphology and crystallinity.^3,4^ In NCs, impurity doping has led to dramatically enhanced photoluminescence^5,6^ (PL), with color tunability across the visible to near-infrared regions of the electromagnetic spectrum,^7^ and with potential applications in display^8^ and biomedical imaging technologies.^9^ Controlled doping of NCs has produced giant induced magnetic moments^10^ with tunable magnetic strength.^11^ Trapping carriers at dopants also yields markedly increased charge-carrier separation lifetimes,^12^ with clear implications for NC photocatalysis.^13^

Recently, the doping of colloidal quasi-2D CdSe nanoplatelets (NPLs) has emerged, yielding distinctive physical properties compared to the spherical NCs.^14–16^ NPLs are an interesting system for exploring impurity doping because their thickness is controlled with atomic precision, resulting in near-homogeneously broadened absorption and PL line widths.^17,18^ Strong exciton quantum confinement perpendicular to the large-area facets of the NPLs^19^ allows for tunable band-edge (BE) exciton emission by controlling the number of NPL monolayers (MLs), while Ag^+^ doping with Cd^2+^ yields subgap PL emission that is continuously tunable from 600 to 900 nm.^14,15^ The dopant PL is attributed to an optical transition between a hole in a midgap Ag^+^ state that lies above the valence band and a confined conduction-band electron state.^7^

Interestingly, simple arguments suggest that both the energy and the lifetime of the dopant PL should depend strongly on the dopant position in the nanoparticle. The radiative decay rate of the Ag^+^ dopant transition is proportional to the square of the electron wave function at the Ag^+^ location.^20^ This wave function is most probably located in the middle of the NPL (along the short thickness direction); therefore, recombination at acceptors near the middle is faster than that near the NPL surface (eq 4). Also, the final state of the recombination process includes an ionized acceptor, which interacts with its mirror image across the dielectric discontinuity at the surface of the NPL, reducing the emission energy by an amount dependent on dopant position. Since dopant PL properties are very strongly related to doping position in the NPL, we propose that dopant PL can be used as a facile tool to map the electron wave function in NPLs.

Here, we demonstrate such a mapping in Ag^+^-doped CdSe NPL, developing an understanding that, importantly, can be generalized across different nanoparticles and a wide variety of impurities. Single-NPL spectroscopy confirms that incorporation of multiple Ag^+^ per CdSe NPLs leads to broad PL line widths with energy-dependent lifetimes, consistent with predictions. Furthermore, single NPLs exhibit dynamic dual band-edge and dopant PL emission even at high impurity concentrations, suggesting strong exciton localization for both states. Finally, we demonstrate that the strong hole localization at Ag^+^ acceptor sites significantly enhances Auger recombination, resulting in high-purity single-photon emission at room temperature.

## Results and Discussion

### Acceptor Depth-Dependent Recombination

Theory can be used to estimate the energy of the dopant PL in Ag^+^ doped CdSe NPLs. A silver ion Ag^+^ substituted for cadmium in bulk CdSe creates a deep acceptor, ${\mathrm{Ag}}_{\mathrm{Cd}}^{-}$ . As shown in Figure 1a, an NC in the ground state (blue) absorbs a photon, generating an electron–hole pair (orange). The hole subsequently localizes on ${\mathrm{Ag}}_{\mathrm{Cd}}^{-}$ , forming ${\mathrm{Ag}}_{\mathrm{Cd}}^{0}$ (green). The zero-phonon line (ZPL) emission energy corresponds to a vibronic transition directly from the equilibrium configuration of the neutral ${\mathrm{Ag}}_{\mathrm{Cd}}^{0}$ to the equilibrium configuration of the ionized ${\mathrm{Ag}}_{\mathrm{Cd}}^{-}$ (Figure 1a, black). The observed PL is red-shifted due to the Stokes shift, which we describe within the Franck–Condon approximation (Figure 1a, red). Using density functional theory, we find the ${\mathrm{Ag}}_{\mathrm{Cd}}^{-}$ binding energy $E_{b}^{A}=$ 0.38 eV and an effective acceptor radius *a*_h_= 0.73 nm (see Methods section and Figure S3 for details), corresponding to a PL energy of 1.21 eV. Moving to NPLs, the upper two curves in Figure 1a are then shifted up by the quantum confinement energy, which is 0.97 eV for 4.5 ML NPLs. Thus, we predict that for BE exciton emission at 2.42 eV, the dopant PL peak is 1.97 eV (630 nm), close to the reported PL emission peaks at ∼660 nm (1.88 eV). Notably, this simple picture does not describe the dopant PL redshift for increasing dopant concentrations observed for NPL systems over the past several years^14,15,21,22^ or explain the exceptionally large ∼350 meV full-width-at-half-maximum (FWHM) PL line width. A more complete model must explain these observations.

**Figure 1:**
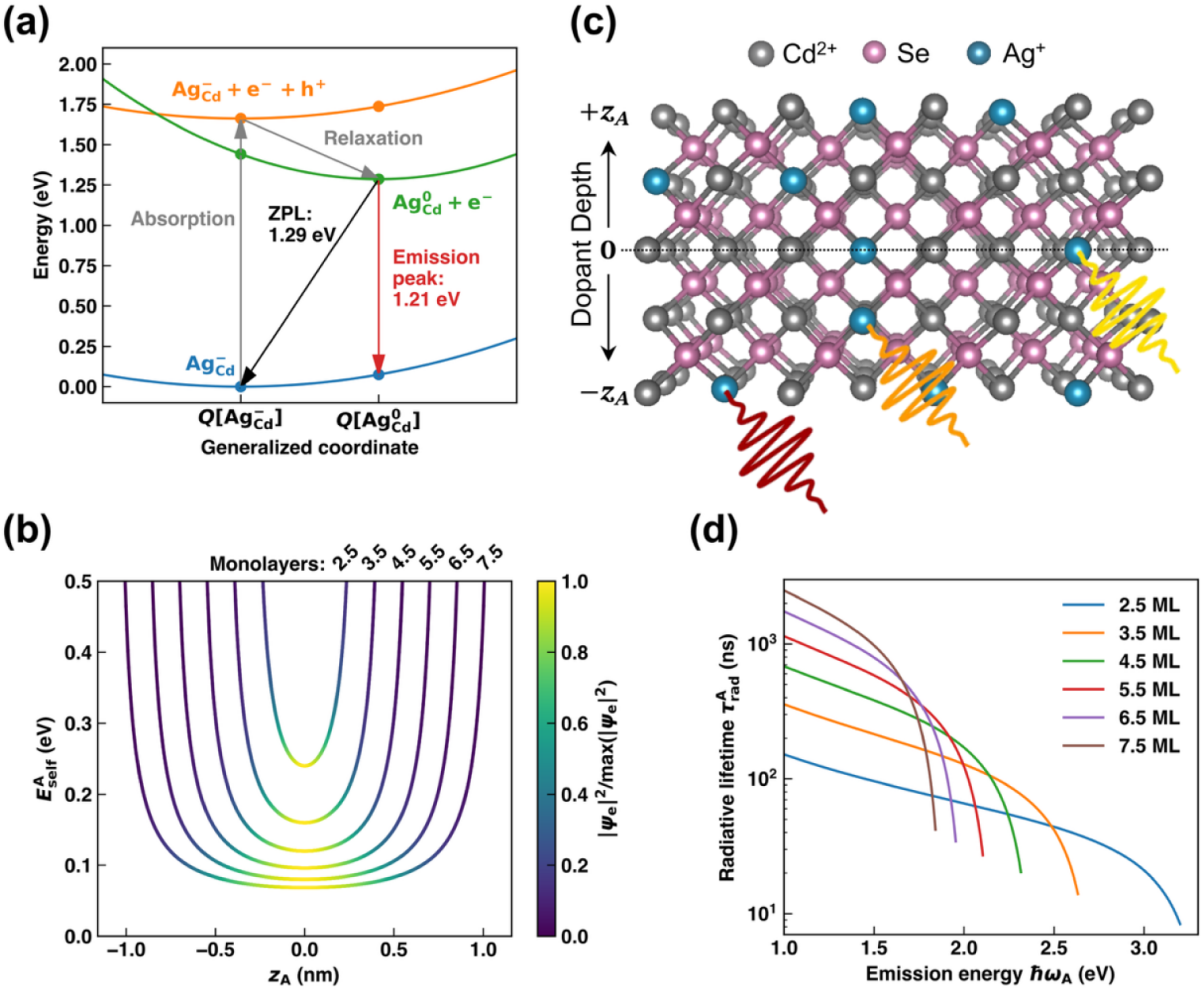
Dopant acceptor position and recombination. (a) Configuration coordinate diagram illustrating the excitation and recombination processes for silver-doped bulk CdSe. The generalized coordinate *Q* interpolates between atomic positions of $Ag_{Cd}^{-}$ and $Ag_{Cd}^{0}$ . Quantum confinement increases the emission energy in thinner NPLs. (b) Self-interaction energy vs depth (*z*_A_) for 2.5–7.5 ML NPLs. The color scale represents the electron wave function probability density. (c) Surface acceptors exhibit lower emission energies (red) due to increased self-interaction compared to acceptors located deeper into the NPL (yellow). (d) Acceptor radiative lifetime ( $\tau_{\mathrm{rad}}^{A})$ vs emission energy. Surface acceptors emit more slowly due to reduced electron density and lower emission frequency.

Since the hole is strongly localized on the acceptor, quantum confinement has a negligible effect on the acceptor binding energy. However, the ionized acceptor experiences electrostatic self-interaction due to the dielectric discontinuity at the nanoplatelet surface.^19,23,24^ We model the NPL as a quantum well with thickness *d* $(-\frac{d}{2}\leq z\leq \frac{d}{2})$ along the (001) direction. The dielectric constant is *ϵ*_in_ inside the NPL and *ϵ*_out_ outside. The self-energy of the acceptor, $E_{\mathrm{self}}^{A}$ , is

1 $$E_{\mathrm{self}}^{A}\left(z_{A}\right)=\frac{e^{2}}{2\epsilon_{in}}\overset{n=\infty }{\underset{n\neq 0,n=-\infty }{\sum }}\frac{q_{n}}{\left|z_{A}-z_{n,A}\right|}$$

where $q_{n}=q_{-n}={[\frac{\kappa -1}{\kappa +1}]}^{|n|},\kappa =\epsilon_{\mathrm{in}}/\epsilon_{\mathrm{out}}$ , *z*_*n*,A_ = (−1)^*n*^ *z*_A_ + *nd*, and *z*_A_ is the *z* coordinate of the acceptor (see Methods section for details). The self-interaction energy of the acceptor is plotted as a function of *z*_A_ for 2.5–7.5 ML NPLs in Figure 1b (e.g., a 3.5 ML CdSe nanoplatelet consists of three layers of Se and four layers of Cd).

The recombination energy of the acceptor is

$$\hbar \omega_{A}\left(d\right)=E_{g}\left(d\right)-E_{b}^{A}+E_{\mathrm{self}}^{e}\left(d\right)-E_{\mathrm{self}}^{A}\left(z_{A}\right)$$ 2

where *E*_g_(*d*) is the energy gap for the various ML NPLs, equal to 3.54, 2.99, 2.68, 2.47, 2.32, and 2.21 eV for 2.5 through 7.5 ML NPLs, respectively,^24^ and $E_{\mathrm{self}}^{e}$ is the self-energy of the electron before recombination. The strong dependence of $E_{\mathrm{self}}^{A}$ on the acceptor depth leads to a broad distribution of recombination energies, with dopants near the surface having more red-shifted PL, as plotted in Figure S4 and illustrated schematically Figure 1c.

In addition to depth-dependent dopant energies, the radiative recombination of an electron onto a deep acceptor, ${\tau_{\mathrm{rad}}^{A}}^{-1}$ , also varies with position (***r***). The recombination rate in any nanostructure is proportional to the square of the overlap integral *K* given by^25^

3 $$K={|\int d^{3}r\Psi_{e}\left(r\right)\Psi_{h}^{A}\left(r\right)|}^{2}$$

where Ψ_e_(**r**) is the electron wave function and $\Psi_{h}^{A}\left(r\right)$ is the wave function of the hole localized on the acceptor.^20^ For 2D NPLs with lateral dimension *L*_*x*_ and *L*_*y*_,

$$\frac{1}{\tau_{\mathrm{rad}}^{A}}=\frac{2\pi }{137}n\omega_{A}\frac{E_{p}}{m_{0}c^{2}}\frac{a_{h}^{3}}{L_{x}L_{y}}|\psi_{e}\left(z_{A}\right){|}^{2}$$ 4

where *E*_P_ is the Kane energy, *n* is the refractive index, *m*_0_ is the free electron mass, *a*_h_ is the effective acceptor radius, $\psi_{e}\left(z\right)=\sqrt{\frac{2}{d+2\delta }}\cos\left(\frac{\pi z}{d+2\delta }\right)$ is the electron wave function well described by the 1D particle-in-a-box model as a function of depth along the NPL thickness, and *δ* is the wave function penetration depth into the surrounding NPL ligand layer (see Methods section – Acceptor Lifetime for more details). Hence, the recombination rate is proportional to $\cos^{2}\left(\frac{\pi z_{A}}{d}\right)$ , leading to much faster recombination near the NPL center (*z*_A_ ∼ 0) compared to the top (*z*_A_ ∼ *d*/2) or bottom (*z*_A_ ∼ −*d*/2) surface. Therefore, the radiative lifetime of the dopant PL effectively maps the cross-sectional dependence of the electron wave function in the nanoplatelet, leading to a strong correlation between the electron wave function probability and dopant PL energy and lifetime (Figure 1b), which is plotted in Figure 1d.

To verify our model, we synthesized both 4.5 and 5.5 ML CdSe NPLs with similar ∼30 nm × 9 nm dimensions^26,27^ and incorporated Ag^+^ dopants via partial cation exchange^14,15^ with the Cd^2+^ atoms (see Methods section). NPLs with low doping (25 Ag/NPL, or 0.5% Cd substitution, as determined from ICP-MS, Table S1) and high doping (>80 Ag/NPL, 1.5%) were synthesized. XPS spectra were acquired to validate the presence of Ag-doping species and their chemical environments. In the survey spectrum, as shown in Figure S14, Ag peaks appear much weaker than the intense signals from carbon, oxygen, cadmium, and selenium, making them difficult to distinguish. In the Ag 3d state spectrum, the Ag 3d_5/2_ binding energy is observed at 368.02 eV, which is in close agreement with the silver selenide binding energy of 367.9 eV, as reported for Ag_2_Se.^28,29^ Moreover, a broad Ag 4p feature in the Se 3d state spectra at ∼59–60 eV falls within the region consistent with the Ag^+^ oxidation state. In addition, the Cd and Se 3d-state peaks align well with their reported values.^30^

As shown in Figure 2a, both samples show BE PL and broad, Stokes-shifted dopant emission. At low doping, the BE PL is dominant, whereas at high doping levels, the dopant PL becomes dominant. We also measured dopant PL lifetimes every 25 nm (with a spectral resolution of 3 nm) across the broad emission line width, which are given by

5 $$\frac{1}{\tau_{\mathrm{avg}}^{A}}=\frac{1}{\tau_{\mathrm{rad}}^{A}}+\frac{1}{\tau_{\mathrm{nr}}^{A}}=\frac{1}{\eta_{\mathrm{QE}}\tau_{\mathrm{rad}}^{A}}$$

where ${\tau_{\mathrm{nr}}^{A}}^{-1}$ is the nonradiative acceptor recombination rate. *η*_QE_ is the quantum efficiency, i.e., the probability of radiative recombination once the hole is localized on the acceptor (distinct from the total PL quantum yield). As shown in Figure 2b for low-doped 4.5 ML NPLs, dopant PL lifetimes steadily increase as the emission energy decreases, which we compared with theory (eqs 4 and 5). Remarkably, with *η*_QE_ as the only fitting parameter, the model predicts the dopant emission energies and their corresponding lifetimes with near-quantitative accuracy. The fitted *η*_QE_ is ∼45% for the 4.5 ML and 69%/55% for the low/high-doped 5.5 ML NPLs. We note that nonradiative pathways may also impact the overall dopant PL lifetimes. Acceptors closer to the NPL surface could be expected to recombine faster with band-edge electrons due to the enhancement of nonradiative recombination by the proximity of surface states. However, in contrast, we find that the measured PL lifetimes are shorter for higher emission energies, which arises from recombination of acceptors in the middle of the NPL. This can be explained by the increase of the electron–hole overlap integral, which is proportional to the square of the electron wave function (Methods section, eq 9) and has a maximum for acceptors in the NPL center, leading naturally to faster decay in the middle.

**Figure 2:**
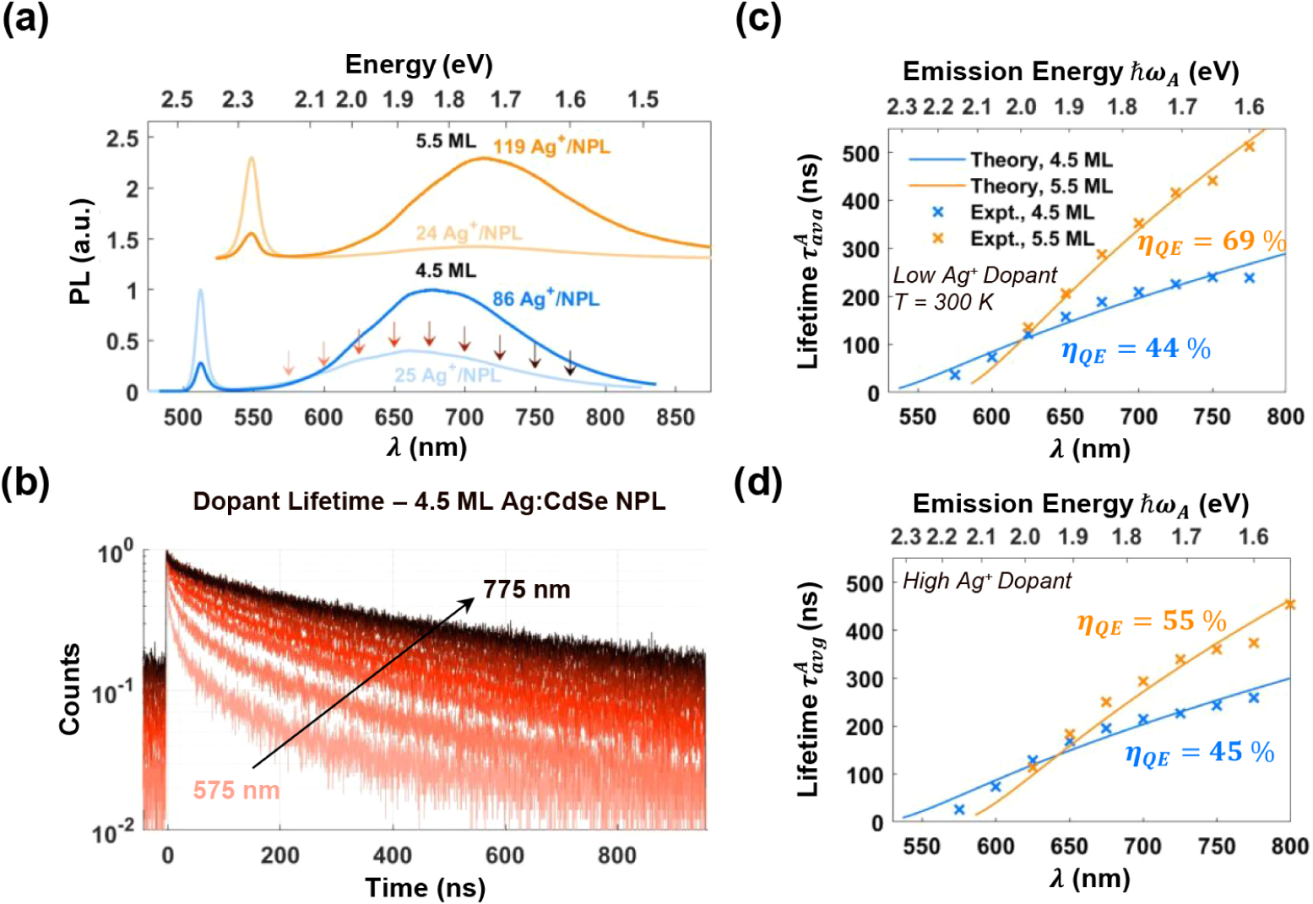
Dopant emission dynamics. (a) Solution photoluminescence spectra of 4.5 and 5.5 ML CdSe NPLs with low and high silver doping. Increased doping quenches band-edge PL and enhances dopant emission. (b) PL decay curves of low-doped 4.5 ML NPLs at 300 K for varying emission wavelengths are color-coded as indicated with arrows in (a). (c-d) Average PL lifetimes vs emission energy compared with the theoretical model (η_QE_ is a fitting parameter) for both 4.5 and 5.5 MLs, at low doping (c) and high doping (d). Dopant acceptor sites closer to the NPL center have higher emission energies and shorter recombination lifetimes.

Electron–phonon coupling (EPC), which depends strongly on temperature, could also play an important role in the overall photophysics of the dopant. For copper-doped CdSe NPLs, Yu et al.^31^ showed that EPC induced by impurity-lattice distortions^32^ leads to a phonon cascade process resulting in broad dopant emission. However, for our Ag:CdSe NPL system, the optical signatures associated with the discrete phonon-replica emission lines were not observed under the weak excitation regime for either ensemble or single-particle measurements. Additionally, upon cooling from 300 to 77 K, the dopant PL FWHM for 4.5 ML (and 5.5 ML) NPLs remains largely unchanged (in fact, it slightly increases), in contrast to the expected line width narrowing from reduced EPC at colder temperatures (394 to400 meV, and 336 to400 meV, respectively, for the low- and high-doped samples, Figure S8). Instead, our measured emission-energy-dependent lifetimes at 77 K are also in excellent agreement with those of the model (Figure S9). Therefore, we attribute the overall broad dopant emission to be largely dominated by the varying acceptor depth positions, as shown in Figure S5, with EPC effects being minor. While energy relaxation via hole hopping/tunneling could potentially lead to changes in emission lifetimes, our relatively low doping concentrations (0.5–1.5% Cd substitution) likely prohibit direct tunneling of strongly localized holes to other acceptors many lattice sites away.

### Single-Particle Spectroscopy: Two-Color Emission

To better understand the relationship between BE and dopant PL emission, we characterized the ensemble BE PL lifetime of 2.42 eV for 4.5 ML NPLs. If NPL doping were heterogeneous, meaning some doped NPLs and some undoped, one would predict two PL lifetimes: one similar to undoped NPLs and one much shorter due to BE PL quenching by the dopant. We observe that, upon dopant incorporation, the BE PL shows shorter lifetimes of 2.8 and 0.24 ns for the low and high dopant concentrations, respectively, versus 3.9 ns for the same undoped NPL sample (Figure S10), with no measurable long-lifetime component. As a result of the BE lifetime shortening, we rule out heterogeneous doping and conclude that the dual BE and PL emission in the ensemble spectra likely arises from all doped NPL samples individually emitting from both BE exciton and acceptor states.

To further investigate the nature of the dual PL emission, we performed single-particle spectroscopic measurements on over 50 individual 4.5 ML Ag^+^-doped NPLs for both low and high dopant concentrations. As shown in Figure 3a, nearly all NPLs exhibit both BE and dopant PL, confirming that all NPLs have Ag^+^ atoms that are substitutionally incorporated. Further, as expected, we find that the average of all single NPL spectra converges to the ensemble spectrum (Figure S11). BE and dopant PL peaks are individually fit to extract their peak emission energies (Figure 3b) and FWHM line widths (Figure 4a). Due to strong exciton confinement, the BE energies are not affected by doping, with an average peak energy of 2.42 eV ± 4 meV (standard deviation), representing a nearly homogeneously broadened line width of ∼40 meV, similar to the undoped sample.

**Figure 3:**
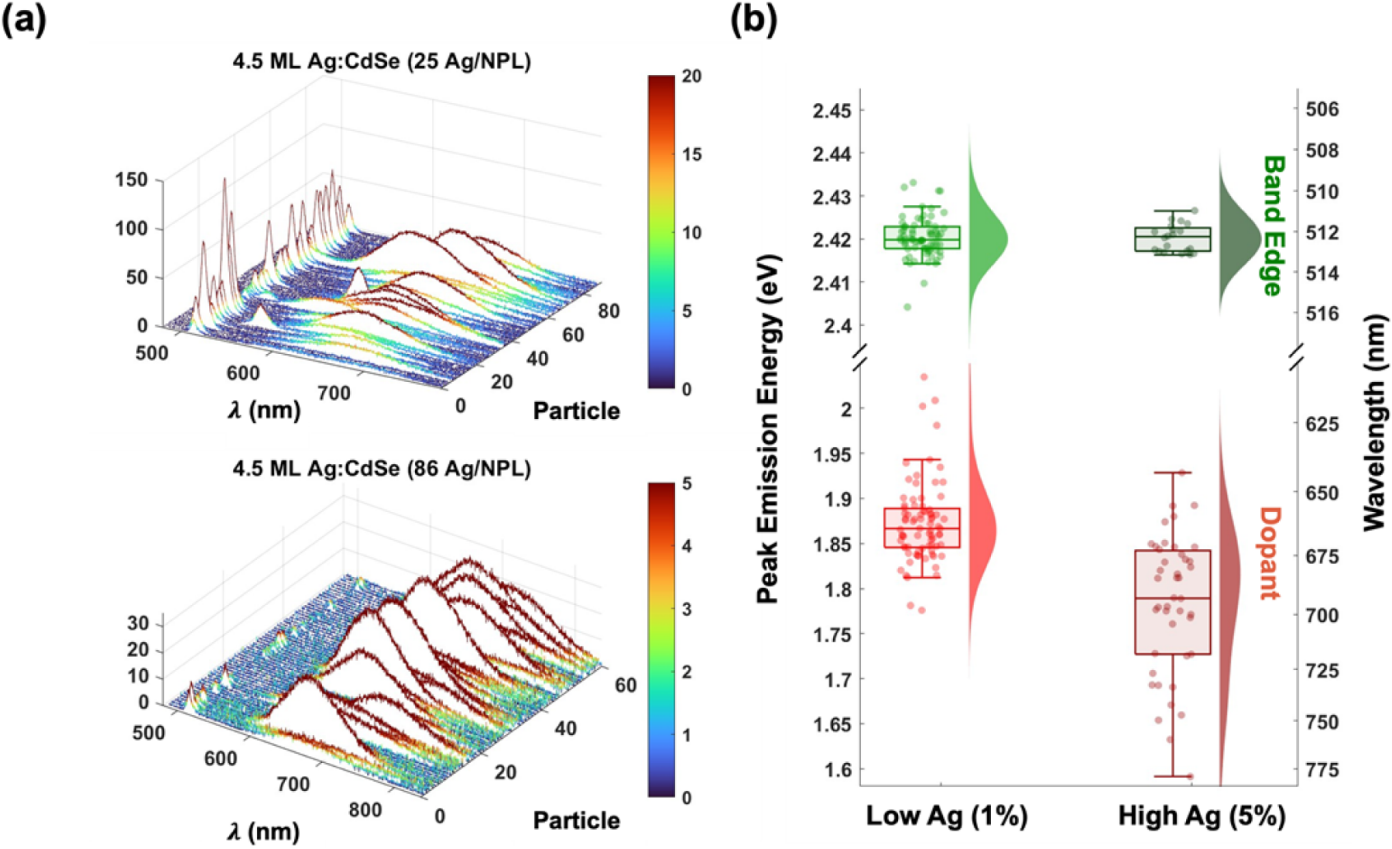
Single Ag^+^-NPL photoluminescence. (a) Single-NPL emission spectra. Nearly all NPLs, both low- and high-doped, exhibit dual PL emission from band-edge and acceptor recombination. (b) PL peak statistics for band-edge and dopant emission in low- and high-doped NPLs. Random dopant incorporation leads to a wide distribution of emission energies, with a higher concentration of red-shifted surface sites at higher dopant densities.

**Figure 4:**
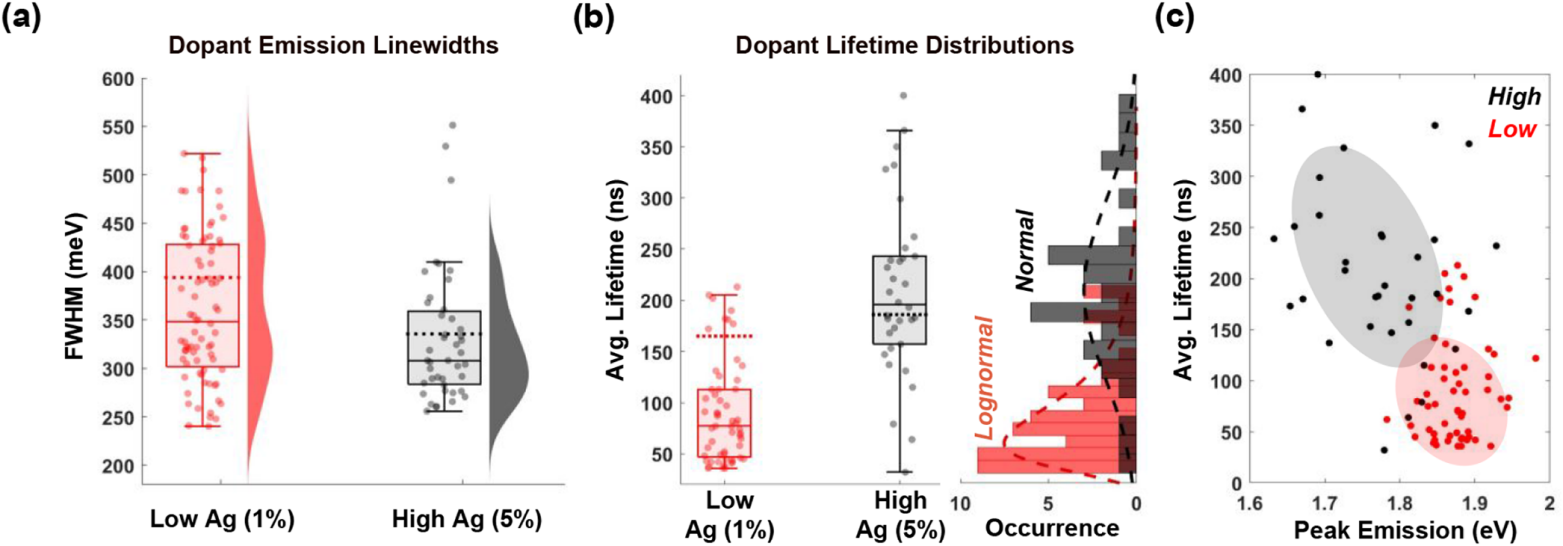
Single-NPL line widths and lifetimes. (a) PL line width distributions for single NPLs from low- and high-doped samples. Line widths of the solution ensemble spectra are shown by dotted lines. Single NPLs and ensembles exhibit similarly broad distributions of emission energies. (b) PL lifetime distributions for single NPLs from low- and high-doped samples. Lifetimes of the solution ensemble spectra are shown as dotted lines. Ensemble lifetimes are much closer to single-NPL lifetimes for high-doped NPLs than for low-doped. (c) Correlating single-NPL peak dopant emission energies with their respective lifetimes confirms the depth-dependent energy-lifetime model. On average, higher doping leads to particles with lower dopant peak emission energies and longer radiative lifetimes, corresponding to sites closer to the NPL surface.

Single NPL dopant PL characteristics can be used to understand the doping process. For example, the large distribution in the peak emission energies across particles, with averages of 1.87 eV ± 45 meV and 1.78 eV ± 77 meV for the low- and high-dopant samples, suggests a large heterogeneity in dopant positions within the NPL (Figure 1c). Single-particle line widths average 345 ± 75 meV across the two samples, similar to the ensemble spectra, confirming that broad dopant emission is intrinsic to the individual doped NPL. The similar individual NPL photoluminescence line width for both low- and high-doping samples suggests substitutional Ag^+^ sites incorporate at all depths. However, high-doped NPLs show a 90 meV redshift of the dopant emission compared to the low-doped samples, suggesting that a larger fraction of Ag^+^ incorporates at the surface. Indeed, dopant aggregation of noble metals on NPL surfaces and corners has been previously reported,^33,34^ and it was also calculated that at high concentrations, dopants can cluster near the surface.^35^

Dopant PL lifetimes from single NPLs also suggest a larger concentration of dopants at the NPL surface for the high-dopant sample. As shown in Figure 4b, we see a wide range of average acceptor recombination lifetimes, ranging from 50 to over 300 ns across all NPLs, indicating heterogeneity in the dopant positions. However, on average, highly doped particles with lower emission energies correspond to a broader distribution of dopant PL line widths, consistent with a larger amount of dopants throughout the NPL thickness, including at the surface. The correlation between peak dopant emission energy and average lifetime persists even at the single‑NPL level (Figure 4c).

We also observed a notable difference between average dopant emission lifetimes measured in solution versus those of single NPLs. For low-doped NPLs, the average solution lifetime (165 ns) was substantially longer than the average single-particle lifetime (90 ns). In contrast, high-doped NPLs show similar lifetimes in solution (186 ns) and individual particles (208 ns). This convergence likely results from the higher concentration of ions (dopants and counterions) at the NPL surface in heavily doped samples, which passivate trap sites that would otherwise introduce nonradiative decay pathways. This passivation effect is particularly important for the dried single NPLs, where surface traps typically have a stronger impact than those in solution.

### Enhanced Photon Antibunching

Compared to QDs, NPLs have reduced nonradiative Auger recombination rates arising from the weak exciton confinement along the extended lateral dimensions.^36^ Reduced Auger recombination promotes multiexciton states, which can lead to multiphoton emission even under relatively low fluence. As a result, NPL biexciton QYs as high as 80% have been reported for core and core–shell CdSe/CdS NPLs.^36,37^ In doped NPLs, however, the stronger hole localization at the acceptor should lead to a more efficient Auger process and thus suppress biexciton QYs. Indeed, other CdSe NPL systems with intentional hole trap states show enhanced single-photon emission.^38^

We characterized photon antibunching in the dopant emission using a Hanbury-Brown-Twiss (HBT) setup, measuring six randomly selected Ag^+^-doped NPLs from both the low and high dopant concentration samples under ambient conditions. To capture any long-term changes arising from the dynamic dopant state, each NPL was irradiated for 75 min. Remarkably, despite the overall broad dopant PL emission, we measured a high degree of single-photon emission purity of 94% (*g* ^2^(0) = 0.06) and 92% (*g* ^2^(0) = 0.08), averaged across all six NPL particles in each sample (Figure 5a, with individual particle measurements shown in Figure S12). In comparison, undoped NPLs exhibited BE single-photon purity of only 67% (*g* ^2^(0) = 0.33) (Figure S13), confirming significant biexciton suppression by the dopants.

**Figure 5:**
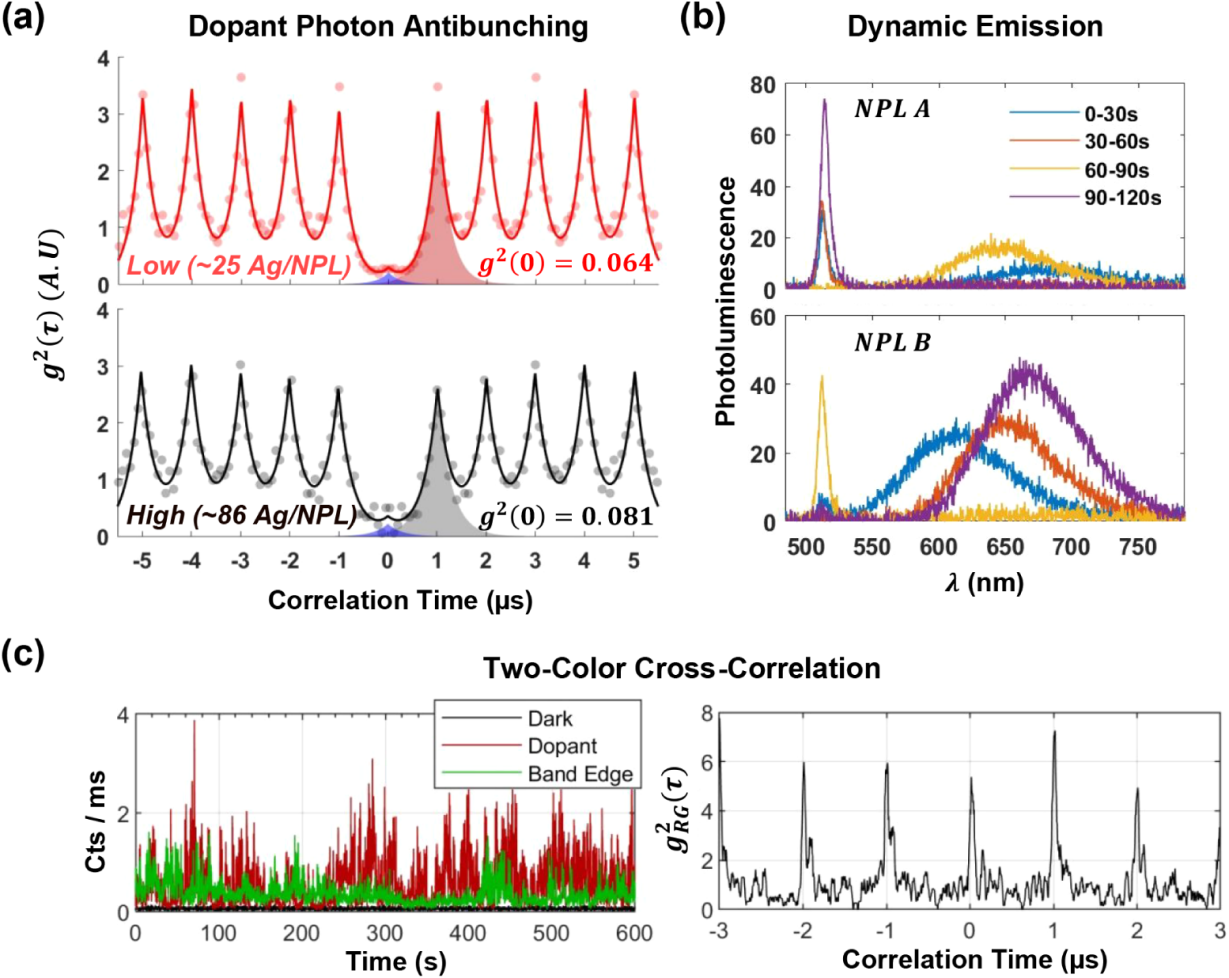
Single-photon emission and spectral dynamics. (a) Photon antibunching (background corrected) from low- and high-doped NPLs (averaged over 6 particles each) demonstrates high single-photon purities: 94% and 92%, respectively. (b) Consecutive averaged 30-s spectra for single NPLs. Spectral wandering reveals dynamic recombination at various acceptor sites. (c) Two-color cross-correlation of band-edge and dopant PL from a single NPL over a 10-min collection window shows uncorrelated emission, indicating independent localization of the two states.

While each NPL contains many randomly positioned acceptor sites, only one is radiatively active during a given pulsed laser excitation cycle. Photon antibunching occurs because electrons are effectively shared in common by all of the acceptors. A deep acceptor with a localized hole creates a very short-range potential, only weakly perturbing the almost-free motion of an electron throughout the entire NPL. If a second electron–hole pair is generated, then the hole will immediately localize on another acceptor, leaving two almost-free electrons. This allows nonradiative Auger recombination involving both electrons and one of the holes. Electron thermalization to the ground state is much faster than electron-acceptor recombination and thus leads to antibunching of the entire acceptor band. Similar single-photon emission was reported by Hinterding et al.^39^ In their case, holes are localized at surface states, but the overall antibunching can be explained by using the same physical mechanism. Over many cycles, different sites emit at varying energies, with each site’s emission energy correlated with its characteristic lifetime. This dynamic behavior is captured in Figure 5b, where consecutive 30-s spectra (each integrating 30 million excitation cycles) reveal dramatic shifts in the peak dopant emission energy. These spectral dynamics confirm the random distribution of recombination locations within the NPL.

Single-NPL spectra reveal that BE PL is sometimes accompanied by dopant emission, while at other times only one state is dominantly emissive. We performed two-color cross-correlation measurements on individual NPLs and found no coupling between the two emission lines (Figure 5c). We hypothesize decoupled, spatially separated exciton recombination regions for the BE and acceptor within a given NPL, consistent with recent reports of BE excitons being confined to small center-of-mass coherence areas^40^ (<10 nm^2^) due to defects or ligand-induced local potential traps.^24^ Furthermore, the fact that quenched BE exciton lifetimes are as short as 240 ps, while dopant lifetimes are 100–1000x longer, suggests that the rapidly flickering BE emission events do not perturb the slower dopant recombination.

## Conclusions

Semiconductor NPLs, a special class of colloidal NCs grown monodisperse with atomic precision, offer tremendous promise as bright, efficient, and color-tunable fluorophores. We present a theoretical model of substitutional Ag^+^ dopants in CdSe NPLs, with emission energies and lifetimes that depend strongly on the acceptor depth. Ensemble and single-particle PL measurements show excellent agreement with theory, verifying the model that dopant recombination sites closer to the NPL surface have lower emission energies and longer PL lifetimes compared to sites located toward the NPL center. Only one doped-acceptor site is radiatively active during each pulsed excitation, resulting in excellent single-photon emission purities over 90% at ambient conditions. We also observe dual emission from individual NPLs, as the large anisotropy and small exciton radius in nanoplatelets allow the formation and recombination of a BE exciton in one region of the platelet, while dopant-bound exciton formation and recombination occur independently in another.

The mechanisms we have explored here are not limited to 2D nanoplatelets: any localized charge in a semiconductor nanostructure will have a similar self-energy due to the dielectric discontinuity at the surface. A random distribution of dopant sites will, therefore, naturally broaden dopant emission, and this phenomenon may be used to map the electron wave function via the radiative lifetime of dopants in any nanocrystal shape. Precise control over the dopant concentration and placement within nanocrystals and nanoplatelets thus offers a powerful tool for tailoring their optoelectronic properties. This capability would advance applications in areas such as donor–acceptor charge transfer,^12^ photocatalysis,^41^ photovoltaics,^42^ and quantum information technology^43^ by enabling the design of more efficient and versatile nanoparticle systems.

## Methods

### Chemicals

Cadmium nitrate tetrahydrate (Cd­(NO_3_)_2_·4H_2_O, > 98%), sodium myristate (C_14_H_27_NaO_2_, ≥99.5%), selenium powder (Se, ≥99.5%, trace metal basis), cadmium acetate dihydrate (Cd­(OOCCH_3_)_2_·2H_2_O, >98%), silver­(I) acetate anhydrous (Ag­(OOCCH_3_), ≥99.9%, trace metal basis), technical grade 1-octadecene (ODE, 90%), technical grade oleic acid (OA, 90%), 3-methyl pentane (≥99%), and 200-proof HPLC-grade ethanol were procured from Sigma-Aldrich. HPLC-grade methanol, hexanes, chloroform, and octane were purchased from Fisher Chemical. HPLC-grade acetonitrile was obtained from VWR Chemicals. All chemicals were used as delivered without further purification.

### NPL Synthesis and Cation Exchange

#### Synthesis of Cadmium Myristate

Cadmium myristate was synthesized according to a previously reported procedure.^14^ 3 g of cadmium nitrate tetrahydrate were dissolved in 200 mL of methanol. Separately, 5 g of sodium myristate were dissolved in 500 mL of methanol using ultrasonication. Using a 50 mL buret, the cadmium nitrate solution was added dropwise into the sodium myristate solution under vigorous stirring to form white precipitates of cadmium myristate. After the complete addition of the 200 mL of cadmium nitrate solution, the reaction was allowed to stir for 2 h. The resulting white precipitate was collected via vacuum filtration using a fine-grit borosilicate glass Büchner funnel. The precipitate was washed three times with an excess of methanol before drying overnight under a vacuum on a Schlenk line. The dried precipitate was collected and kept under vacuum in the antechamber overnight before transferring and storing in a N_2_-controlled glovebox.

#### Synthesis of CdSe Nanoplatelets

##### 4.5 Monolayer

The 4.5 ML nanoplatelets were synthesized guided by a previously reported procedure.^26^ In a three-neck 100 mL round-bottom flask, 180 mg of cadmium myristate, 30 mg of selenium powder, and 15 mL of 1-octadecene were loaded. The contents of the flask were purged with N_2_ and then degassed at room temperature under vacuum on a Schlenk line. The temperature was then increased to 120 °C, and the reaction was kept under vacuum for 1 h. The flask was then returned to N_2_, and the temperature was set to 240 °C with active refluxing. At exactly 210 °C, the septum from one neck of the flask was removed under low N_2_ flow, and 100 mg of cadmium acetate dihydrate was swiftly injected into the reaction. The neck was closed, and once the temperature reached 240 °C, the reaction was allowed to proceed for exactly 8 min. The reaction was then quenched using an air gun and further cooled with a water bath at 190 °C. 2 mL of oleic acid were injected at 160 °C, and once the reaction reached room temperature, 15 mL of hexanes were injected into the solution. The solution was then split into four 50 mL Falcon tubes and centrifuged at 3000 rpm for 10 min. The supernatant was discarded, and the pellet was redispersed in 12 mL of hexanes. The solution was then allowed to rest in the dark overnight, and the following day it was centrifuged at 6000 rpm for 15 min. The resulting 4.5 ML CdSe nanoplatelet solution was stored in an airtight, dark environment, and the pellet was discarded.

##### 5.5 Monolayer

The 5.5 ML nanoplatelets were synthesized guided by a previously reported method.^27^ 340 mg of cadmium myristate and 14 mL of 1-octadecene were loaded into a 100 mL 3-neck round-bottom flask. This mixture was degassed under vacuum at room temperature for 1 h. The flask contents were then heated to 250 °C under a N_2_ controlled environment. Once stabilized at 250 °C, a freshly sonicated solution of 24 mg of selenium powder in 1 mL of 1-octadecene was quickly injected into the flask. The temperature dropped to 245–248 °C depending on the experimental setup. After 110–120 s, once the temperature attains 250 °C again, 140 mg of cadmium acetate dihydrate were introduced by opening one neck of the flask under a low N_2_ flow. The particles were then allowed to grow at 250 °C for 10 min, following which the reaction was quickly quenched by the addition of 2 mL of oleic acid and further cooled to room temperature with an air gun and water bath. For the purification of 5.5 ML NPLs, 15 mL of hexanes and 10 mL of ethanol were directly added to the solution, which was then split into four 50 mL Falcon tubes and centrifuged at 4000 rpm for 15 min. The resulting precipitate was redispersed in a total of 12 mL of hexanes and recentrifuged at 4000 rpm. The supernatant containing 5.5 ML of NPL was collected and stored in a dark environment.

#### Ag^+^ Doping of CdSe Nanoplatelets

The silver doping procedure was adapted from previously reported literature with a few modifications.^14,15^ A silver acetate precursor solution of 0.006 M concentration was prepared by freshly dissolving the salt in a 5:2 methanol:nanopure water solvent system. The concentrations of 4.5 and 5.5 ML nanoplatelets were determined by UV–vis absorption values using the extinction coefficient ε = 3.19 × 10^7^ cm^–1^ M^–1^ at the 2.42 eV hh-e peak, and 2.45 × 10^7^ cm^–1^ M^–1^ at the 2.24 eV hh-e peak for a surface area of ∼260 nm^2^ and ∼275 nm^2^, respectively, as reported by Yeltik et al.^44^ The nanoplatelet stock solution was diluted 7x with hexanes, and the volume of the dopant solution was calculated for the desired dopant percentage loading as a fraction of cadmium ions per nanoplatelet. The silver solution was added dropwise to the nanoplatelet solution at room temperature under vigorous stirring at 1000 rpm, and the reaction was allowed to proceed for 1 h, after which the solution was allowed to rest in a dark, air-free environment for 2 days to achieve equilibrium before collecting spectroscopic measurements.

### Characterizing NPL Particle Morphology

#### Transmission Electron Microscopy (TEM)

Dilute (100–200 times) solutions of nanoplatelet samples were drop-cast onto ultrathin carbon film supported by a lacey carbon film on 400-mesh copper grids from Ted Pella, Inc. Transmission electron microscopy (TEM) micrographs were collected using an FEI Tecnai F20 field emission microscope with a 200 kV accelerating voltage, maintained by the University of Rochester Integrated Nanosystems Center. Nanoplatelet size statistics were recorded using the National Institutes of Health ImageJ software.

#### Inductively Coupled Plasma-Mass Spectrometry (ICP-MS)

The nanoplatelets were subjected to multiple rounds of washing with antisolvents before ICP measurements. For core NPLs, in a 15 mL Falcon tube, ethanol was added to the NPL solution in a 1:1 ratio. The NPLs were centrifuged at 6000 rpm for 15 min and then redispersed in the initial volume of hexanes. This was followed by three consecutive rounds of washing, with one part of acetonitrile added to one part of the NPL solution, followed by one part of chloroform addition, centrifugation at 6000 rpm, and redispersion in hexanes. The same process was repeated for Ag^+^-doped NPLs, with one EtOH wash and two rounds of MeCN: CHCl_3_ washings. For ICP, 0.5 mL of concentrated nitric acid (ultrapure) was added to a weighed sample and placed on a hot block for one h at 95 °C. After cooling, the samples were transferred to a 50 mL polypropylene tube and diluted to 250 mL. The measurements were collected using a NexION 2000C ICP mass spectrometer.

#### X-ray Photoelectron Spectroscopy (XPS)

For the preparation of XPS samples, silicon wafers were cleaned with acetone, sonicated, and rinsed again with acetone. Silver-doped nanoplatelets were washed twice with ethanol in a 1:1 volumetric ratio. The washed oleic acid-capped nanoplatelets were deposited on silicon wafers and dried overnight under ambient conditions for XPS analysis. XPS spectra were collected on a Kratos AXIS Ultra DLD spectrophotometer with an incidence angle of 60° and a takeoff angle of 90°. The hybrid lens spot size was approximately 0.7 mm × × 0.3 mm (0.21 mm^2^). Charge neutralization was used to minimize the charge accumulation of the sample during data collection. Analyses of XPS spectra were performed using CasaXPS software (Casa Software Ltd.), and peak assignments (e.g., CdSe, Ag_2_Se) were made based on the comparison of the measured binding energy with literature values.^28^ Spectral baselines were fit with Shirley background algorithms and subtracted. Peaks were fit with pseudo-Voigt curves (30% Lorentzian) using the minimum number of peaks required to provide an adequate fit to the spectrum. All peaks for a given high-resolution spectrum were constrained to have a consistent full width at half-maximum (FWHM). Spin–orbit splitting area ratios were constrained to 2:3 for d-subshells. Spin–orbit splitting peak spacings were not used as constraints but were confirmed to be approximately equal to literature values.^28^ These splittings were Ag 3d = 6.07 eV (lit. 6.0 eV), Cd 3d = 6.75 eV (lit. 6.8 eV), and Se 3d = 0.88 eV (lit. 0.86 eV). Relative sensitivity factors (RSFs) for each element were calculated in CasaXPS by using theoretical Scofield values. Calculated RSFs include C 1s 1.0; O 1s 2.93; Ag 3d 18.04; Cd 3d 20.22; Se 3d 2.18.

### Ensemble Spectroscopy

#### UV–VIS and Fluorescence Spectrophotometry

UV–vis absorption spectra were collected by using a PerkinElmer Lambda 950 UV/vis spectrophotometer. Photoluminescence (PL) measurements were obtained using an in-house-built fluorometer setup with a 450 W xenon arc lamp source coupled to an excitation SpectraPro 150 monochromator. A photomultiplier tube (PMT) was used for PL detection, coupled to an emission SpectraPro 300i monochromator. All samples were collected at an excitation wavelength of 430 nm, with data recorded every 1 nm at a 150 ms integration time. All PL measurements were corrected for detector efficiency.

300 K: Measurements were collected using a 1 cm path-length Infrasil cuvette, with all samples diluted in hexanes.

77 K: All measurements were collected using Wilmad precision NMR Class A glass tubes with a 0.38-mm thin wall and an outer diameter of 5 mm, suspended in a coldfinger dewar . All samples were diluted in 3-methylpentane.

#### Relative Quantum Yield (QY) Measurements

QY measurements were collected relative to Coumarin 540A/Coumarin 153 from Luxottica Exciton. A 0.0003 M solution in ethanol at an optical density of ∼0.04 at 422 nm in a 1 cm path length Infrasil cuvette was used as a reference.

#### Energy-Lifetime Measurements

For wavelength-dependent lifetime measurements, solutions were prepared in glass NMR tubes and placed in a coldfinger dewar for both 300 and 77 K (with liquid nitrogen) measurements. The sample was excited with a defocused widefield 485 nm laser spot (1 MHz) via a 10x microscope objective in an epifluorescence configuration. Broad dopant PL was collected via the same objective and filtered using a spectrograph grating with an exit slit (∼3 nm bandwidth) and focused onto an avalanche photodiode with a time correlator.

### Single-Particle Photoluminescence Spectroscopy

For sample preparation, fresh stock NPL solution (undoped and doped) in hexanes was diluted (∼10^6^×) in 90%/10% hexane/octane solvent to reduce the evaporation rate. Subsequently, under ambient conditions, 10–15 μL of the diluted solution was drop-casted onto a clean #1.5 25 mm coverslip and immediately covered. After 2 min of drying, the coverslip was sealed onto a silicone chamber (Grace Bio-Laboratories) to mitigate excess humidity exposure during long data acquisitions. As the extreme dilutions are not stable in the long term, fresh dilutions were prepared immediately prior to coverslip deposition.

Single NPL measurements were acquired using a home-built microscopy setup consisting of an inverted epifluorescence microscope (Nikon TE-2000U) with 485 nm pulsed laser excitation operating at a 1 MHz repetition rate (PicoQuant D-C-485). The laser is focused onto the coverslip with a 100×/1.3 NA oil-immersion objective (Nikon Plan Fluor), giving an ∼1 μm spot size. A defocusing lens is used prior to the objective (excitation path) for widefield imaging of the NPLs with a ∼40 μm × 40 μm illumination spot with a target density of ∼10–20 particles per widefield image. A custom automated single-particle scanning system was utilized with an XY piezo stage (Mad City Laboratories NanoH100), particle identification (MATLAB), and instrument synchronization (LabVIEW). On one side of the microscope exit port, particle spectra were acquired (120 s average) with a 150 g/mm grating using a spectrometer (Acton SpectraPro-2500i) and a liquid nitrogen-cooled CCD (Princeton Instruments LN400BR, 1340 × 400). On a different exit port, PL lifetimes and photon antibunching measurements were acquired using APDs (Micro Photon Devices, PDM50) with appropriate emission filters and a time-tagging correlator (PicoQuant PicoHarp300). The Hanbury Brown-Twiss setup was configured with a 50/50 nonpolarizing beam splitter for photon correlations and a 530 nm dichroic mirror (Thorlabs) for two-color cross-correlation measurements. For both low and high dopant NPL single-particle data sets, laser fluence is fixed to be ∼3 W/cm^2^ (1 MHz excitation), with changes in fluence possibly arising from small misalignments of particles in the laser excitation volume. For photon antibunching curves averaged across 6 particles (7.5 h total), photon correlations per particle were measured across a 75 min integration time. For undoped NPLs, photon antibunching is collected with a 5 MHz laser repetition under a similar fluence, with 30 min integration per particle. PL intensity blinking traces and *g* ^2^(0) < 0.5 are used to confirm single-particle measurements.

For data analysis, spectra are corrected for the grating and CCD detector efficiencies. Individual particle lifetimes are extracted using exponential tail fitting of the raw time-correlated single-photon counting (TCSPC) curves (PicoQuant SymPhoTime 64) and reported as amplitude-weighted averages for multiexponential fits (see Supporting Information for more details). Pulsed photon antibunching curves are also extracted from the same software with no time-gating. The long photon-correlation acquisition times for the dopant emission result in the accumulation of dark/stray-light counts. We attribute the high background in the antibunching traces to a combination of APD dark counts accumulated over long data collections and potentially weak, delayed dopant emission outside the 1 μs pulsed excitation cycles. To properly account for this, we fit the raw traces with a two-sided exponential function to extract and report the background-corrected *g* ^2^(0) values (see Supporting Information for more details).

### DFT Calculations

DFT calculations used the VASP code with PAW pseudopotentials and a plane-wave cutoff of 500 eV. The HSE hybrid functional was employed with a 26.6% mixing parameter, tuned to match the band gap and lattice constant of bulk CdSe. van der Waals interactions were included using the DFT-D3 method of Grimme with zero damping, using the HSE-tuned parameters *S*_8_ = 0.722 and *S*_*R*,6_ = 1.217. Defect supercells had 64 atoms and sampled the Brillouin zone at the special (1/4, 1/4, 1/4) *k*-point, and the FNV correction scheme^45^ was used to correct for spurious periodic-image interactions in the charged defect cells.

The formation energy *E* ^f^ of a silver atom substituted on a Cd site (*A*g_Cd_) is

6 $$E^{f}\left[{\mathrm{Ag}}_{\mathrm{Cd}}^{q}\right]=E_{\mathrm{tot}}\left[{\mathrm{Ag}}_{\mathrm{Cd}}^{q}\right]-E_{\mathrm{tot}}\left[\mathrm{bulk}\right]-\mu_{\mathrm{Ag}}+\mu_{\mathrm{Cd}}+qE_{F}$$

where $E_{\mathrm{tot}}\left[{\mathrm{Ag}}_{\mathrm{Cd}}^{q}\right]$ is the DFT total energy of a CdSe supercell with a substitutional Ag atom and charge *q*, *E*_tot_[bulk] is the total energy of the pristine supercell, *μ*_Ag_ and *μ*_Cd_ are the chemical potentials of Ag and Cd, respectively, and *E*_F_ is the Fermi level, which can be treated as a variable running from the valence-band maximum to the conduction-band minimum. The chemical potentials (which reflect growth conditions) are referenced to the elemental phases and are limited by the requirement that CdSe is stable: μ_Cd_ + μ_Se_ = Δ*H*_f_[CdSe]. There are two limiting cases: cadmium-rich (*μ*_Cd_ = 0) and selenium-rich (*μ*_Se_ = 0). The silver chemical potential is limited by the constraint that alternate phases must not form: CdAg_3_, Cd_8_Ag_5_, and Ag_2_Se were considered. The formation energies of these impurities are plotted in the Supporting Information (Figure S3).

The binding energy of a deep acceptor is equal to the thermodynamic transition level between the neutral and negative charge states:^46^

7 $$E_{b}^{A}=\epsilon \left(0/-\right)=E^{f}\left[{\mathrm{Ag}}_{\mathrm{Cd}}^{-};E_{F}=0\right]-E^{f}\left[{\mathrm{Ag}}_{\mathrm{Cd}}^{0};E_{F}=0\right]$$

Here $E^{f}\left[{\mathrm{Ag}}_{\mathrm{Cd}}^{q};E_{F}=0\right]$ is the formation energy of the substitutional silver with charge *q* when the Fermi level *E*_F_ is at the valence band maximum.

The effective acceptor radius is calculated based on the relation

8 $$E_{b}^{A}=\frac{\hbar^{2}}{2m_{h}a_{h}^{2}}$$

#### Acceptor Lifetime

The lifetime of an exciton is given by^20^

9 $$\frac{1}{\tau }=\frac{1}{3}\frac{n\omega }{137}\frac{E_{p}}{m_{0}c^{2}}{|K|}^{2}$$

where *n* is the refractive index, *ω* is the frequency of the emitted light, *E*_p_ is the Kane energy, *m*_0_ is the free electron mass, and *c* is the speed of light. The overlap integral *K* is given by

$$K=\int d^{3}r\Psi_{e}\left(r\right)\Psi_{h}^{A}\left(r\right)$$ 10

where Ψ_e_(**r**) is the electron wave function and $\Psi_{h}^{A}\left(r\right)$ is the wave function of the hole localized on the acceptor. Because the deep acceptor state is localized in a radius much smaller than that of the nearly free electron, we make the approximation

11 $$K=\Psi_{e}\left(r_{A}\right)2\sqrt{3\pi }a_{h}^{3/2}$$

Here, **r**_A_ is the position of the acceptor, and *a*_h_ is the effective acceptor radius. Analysis of the DFT-calculated nanoplatelets in ref 24 shows that the ligand fluctuations mainly act on the hole, and that the electron is largely unaffected. Therefore, we will assume the wave function of the free electron is spread across the whole nanoplatelet:

12 $$\Psi_{e}\left(x,y,z\right)=\frac{2\sqrt{2}}{\sqrt{L_{x}L_{y}d}}\cos\left(\frac{\pi x}{L_{x}}\right)\cos\left(\frac{\pi y}{L_{y}}\right)\cos\left(\frac{\pi z}{d}\right)$$

13 $$K^{2}=96\pi \frac{a_{h}^{3}}{L_{x}L_{y}d}\cos^{2}\left(\frac{\pi x_{A}}{L_{x}}\right)\cos^{2}\left(\frac{\pi y_{A}}{L_{y}}\right)\cos^{2}\left(\frac{\pi z_{A}}{d}\right)$$

Thus, we obtain the acceptor radiative lifetime $\frac{1}{\tau_{\mathrm{rad}}^{A}}$ :

14 $$\frac{1}{\tau_{\mathrm{rad}}^{A}}=\frac{32\pi }{137}n\omega_{A}\frac{E_{p}}{m_{0}c^{2}}\frac{a_{h}^{3}}{L_{x}L_{y}d}\cos^{2}\left(\frac{\pi x_{A}}{L_{x}}\right)\cos^{2}\left(\frac{\pi y_{A}}{L_{y}}\right)\cos^{2}\left(\frac{\pi z_{A}}{d}\right)$$

Averaging *x*_A_ and *y*_A_ over the nanoplatelet, we obtain,

15 $$\frac{1}{\tau_{\mathrm{rad}}^{A}}=\frac{8\pi }{137}n\omega_{A}\frac{E_{p}}{m_{0}c^{2}}\frac{a_{h}^{3}}{L_{x}L_{y}d}\cos^{2}\left(\frac{\pi z_{A}}{d}\right)$$

The wave function leaks into the ligand layer, so the rate does not go exactly to zero at the surface. We take this into account by defining the leakage distance δ, so the effective width of the platelet becomes *d* + 2δ. Then,

16 $$\frac{1}{\tau_{\mathrm{rad}}^{A}}=\frac{8\pi }{137}n\omega_{A}\frac{E_{p}}{m_{0}c^{2}}\frac{a_{h}^{3}}{L_{x}L_{y}\left(d+2\delta \right)}\cos^{2}\left(\frac{\pi z_{A}}{d+2\delta }\right)$$

We will use *n* = 2.47, *E*_p_ = 17.5 eV, *m*_h_ = 0.19*m*_0_, *L_x_* = 10 nm, *L*_*y*_ = 10 nm, and thickness-dependent *m*_e_ from ref 24. The wave function penetration depth, δ, into the ligand layer is fixed to be 0.55 Å and 0.65 Å for the 4.5 and 5.5 ML NPLs, respectively.

#### Acceptor Self-Energy

Since the hole is strongly localized on the acceptor, its energy is not strongly affected by quantum confinement. However, the charged acceptor ${\mathrm{Ag}}_{\mathrm{Cd}}^{-}$ state does experience an electrostatic self-interaction energy due to the dielectric discontinuity at the nanoplatelet surface. We describe the self-interaction using the image charge method of Hanamura et al.^47^ In a quantum well (*d*/2 ≤ *z* ≤ *d*/2) with dielectric constant *ε*_in_, which is sandwiched within a medium that has a larger energy gap and a smaller dielectric constant *ε*_out_, the Coulomb interaction between an electron and hole can be written in terms of the relative radial coordinate of the electron and hole in the plane, *ρ*, and the z-coordinates *z*_e(h)_ of the electron and hole, as

17 $$V_{\mathrm{eh}}^{3d}\left(\rho ,z_{e},z_{h}\right)=-\frac{e^{2}}{\varepsilon_{i}}\overset{n=\infty }{\underset{n=-\infty }{\sum }}\frac{q_{n}}{\sqrt{\rho^{2}+{\left(z_{e}-z_{h,n}\right)}^{2}}}$$

where *q*_*n*_ = *q*_–*n*_ = [(*k* – 1)/(*k* + 1)]^|*n*|^, *k* = *ε*_in_/*ε*_out_, and *z*_h,*n*_ = (−1)^*n*^ *z*_h_ + *nd*. For CdSe nanoplatelets, we use *ε*_in_ = 6.1 and *ε*_out_ = 2.25. The self-interaction potential is

$$V_{s}\left(z_{A}\right)=-{\frac{1}{2}\left(V_{eh}^{3d}\left(\rho ,z_{e},z_{h}\right)-\frac{e^{2}}{\varepsilon \left|r_{e}-r_{h}\right|}\right)|}_{z_{A}=z_{e}=z_{h},\rho =0}$$ 18

The self-energy of the positive charge of acceptor is given by eq 1 from the main text. The self-energy for the delocalized electron can be written similarly:

$$E_{\mathrm{self}}^{e}\left(d\right)=\frac{1}{d}\frac{e^{2}}{\varepsilon_{\mathrm{in}}}\int_{-d/2}^{d/2}dz_{e}\left[\cos^{2}\left(\frac{\pi z_{e}}{d}\right)\overset{n=\infty }{\underset{n\neq 0,n=-\infty }{\sum }}\frac{q_{n}}{\left|z_{e}-z_{n,e}\right|}\right]$$ 19

where *z*_*n*,e_ = (−1)^*n*^ *z*_e_ + *nd*. The electron self-energy in 2.5 through 7.5 monolayer nanoplatelets is 0.28, 0.19, 0.14, 0.11, 0.1, and 0.08 eV, respectively. The acceptor self-energy at the center (*z*_A_ = 0) is 0.24, 0.16, 0.12, 0.10, 0.08, and 0.07 eV, respectively, smaller than that for the delocalized electron. However, it increases rapidly as *z*_A_ approaches the platelet surface at *z*_A_ = ±*d*/2.

Note that, when describing the emission energy of the acceptor, we neglect the self-interaction of the neutral acceptor due to the strong localization of the hole wave function on the acceptor. We also neglect self-interaction in calculations of the fundamental energy gap, as experimental optical energy gaps in NPLs are well described considering self-interaction.

## Supplementary Material



## Data Availability

The data that support the findings of this study are openly available in OSF at DOI:10.17605/OSF.IO/XBR6D. A preprint version of this manuscript has been previously submitted to the ChemRxiv repository: Amin M, Awan F, Swift MW, Girten W, Erwin SC, Efros Al. L., et al. Depth-Dependent Emission from Silver Dopants in Single CdSe Nanoplatelets. **2025**, *ChemRxiv*. DOI: 10.26434/chemrxiv-2025-b9m9b (accessed Oct 23, 2025).

## Notes

Provided in a separate document containing supplemental figures and further details on spectral and antibunching measurements, along with the theoretical modeling of various monolayer NPLs (PDF)

## Abbreviations

NC: nanocrystal
PL: photoluminescence
CdSe: cadmium selenide
NPL: nanoplatelet
BE: band-edge
ML: monolayer
ZPL: zero-phonon line
fwhm: full-width-at-half-maximum
XPS: X-ray photoelectron spectroscopy
QE: quantum efficiency
QY: quantum yield
EPC: electron–phonon coupling
HBT: Hanbury Brown-Twiss
TEM: transmission electron microscopy
ICP-MS: inductively coupled plasma-mass spectroscopy
TCSPC: time-correlated single-photon counting
APD: avalanche photodiode
CCD: charge-coupled device
DFT: density functional theory
