# Supplementary material for: Depth-Dependent Emission from Silver Dopants in Single CdSe Nanoplatelets
Source: ACS Nano. 2026 Feb 4;20(6):4677–88. doi: 10.1021/acsnano.5c11745 (PMC12918723; doi:10.1021/acsnano.5c11745)
Supplement: Supplementary file 1 [file nn5c11745_si_001.pdf]

Supplementary Information:

Depth-Dependent Emission from Silver Dopants in Single CdSe Nanoplatelets

*Mitesh Amin,[1†] Farwa Awan,[2†] Michael W. Swift,[3] William Girten,[2] Sean W. O'Neill,[4] Steven C. Erwin,[3] Alexander L. Efros,[3] Todd D. Krauss[2,1]**

†M.A. and F.A. contributed equally to this paper

**Corresponding author: Todd D. Krauss (todd.krauss@rochester.edu)*

[1]The Institute of Optics, University of Rochester, Rochester, NY 14627, USA

[2]Department of Chemistry, University of Rochester, Rochester, NY 14627, USA

[3]Center for Computational Materials Science, U.S. Naval Research Laboratory, Washington D.C. 20375, USA

[4]University of Rochester Integrated Nanosystems Center (URnano), University of Rochester, Rochester, NY 14627, USA

Table of Contents

Fig. S1. TEM of CdSe NPLs 3

Table S1. Inductively Plasma Coupled Mass Spectrometry (ICP-MS) 3

Fig. S2. Ensemble characterization of NPL samples (300 K) 4

Fig. S3. Ag defect formation energies as a function of Fermi level in bulk CdSe 4

Fig. S4. Correlation between emission energy and radiative rate. 5

Fig. S5. Dopant Emission Energies (Experiment vs. Model) 5

Fig. S6. Ensemble 4.5 ML CdSe NPL PL lifetimes vs. dopant emission wavelength 6

Table S2. Fitted Energy-Lifetime for 4.5 ML Ag-NPLs (300 K, 77 K) 7

Table S3. Fitted Energy-Lifetime for 5.5 ML Ag-NPLs (300 K, 77 K) 8

Fig. S7. Ensemble 4.5 & 5.5 ML CdSe NPL average PL lifetimes 9

Fig. S8. Dopant Emission Linewidth (300 K vs 77 K) 10

Fig. S9. Lifetime-energy dopant dynamics (77 K) 10

Fig. S10. Ensemble 4.5 ML CdSe NPL PL lifetimes (300 K) 11

Table S4. Fitted Lifetimes for Band Edge vs. Dopant 11

Fig. S11. Single particle vs. ensemble spectra 12

Fig. S12. Photon antibunching from low-doped 4.5 ML CdSe NPLs 13

Fig. S13. Photon antibunching from undoped 4.5 ML CdSe NPLs 14

Fig. S14. X-ray Photoelectron Spectrum from Ag-Doped 4.5 ML CdSe NPLs 14

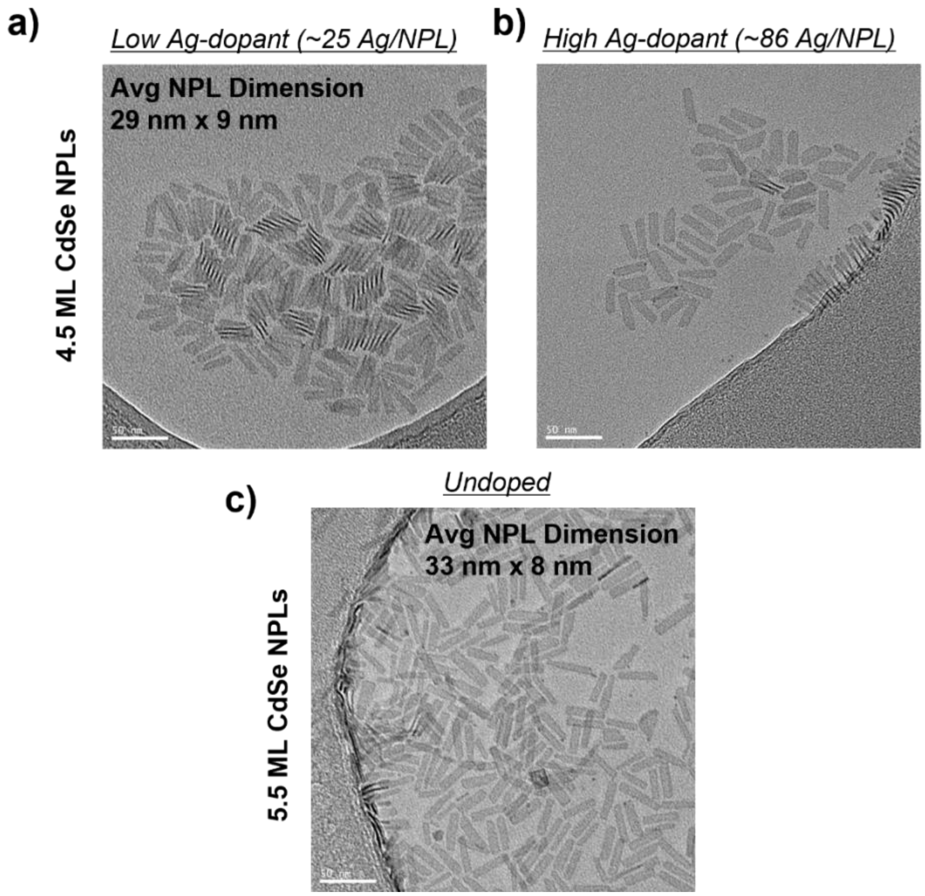

Figure S1. TEM of CdSe NPLs. (a) Transmission electron micrographs of low (~25 Ag/NPL) and **(b)** high (~86 Ag/NPL) doped 4.5 ML CdSe NPLs from the same undoped NPL stock (29 nm x 9 nm) sample solution. **(c)** 5.5 ML CdSe NPLs are synthesized with similar surface areas (33 nm x 8 nm) and silver-doped with similar low and high concentrations. No changes to the particle morphology is observed on TEM post partial cation-exchange doping reaction. Scale bar is 50 nm.

Table S1. Inductively Coupled Plasma Mass Spectrometry (ICP-MS).

	Surface Area (nm^2)	Ag: Cd % Injected	Ag: Cd% Achieved	N_{Ag+}/NPL	Ag^+/nm^2	PLQY (%)
**4 ML Low**	257.75	1%	0.45	25.33	0.098	27
**4 ML High**		5%	1.52	85.55	0.33	9
**5 ML Low**	274.25	1%	0.32	24.1	0.088	20
**5 ML High**		5%	1.60	119.2	0.43	10

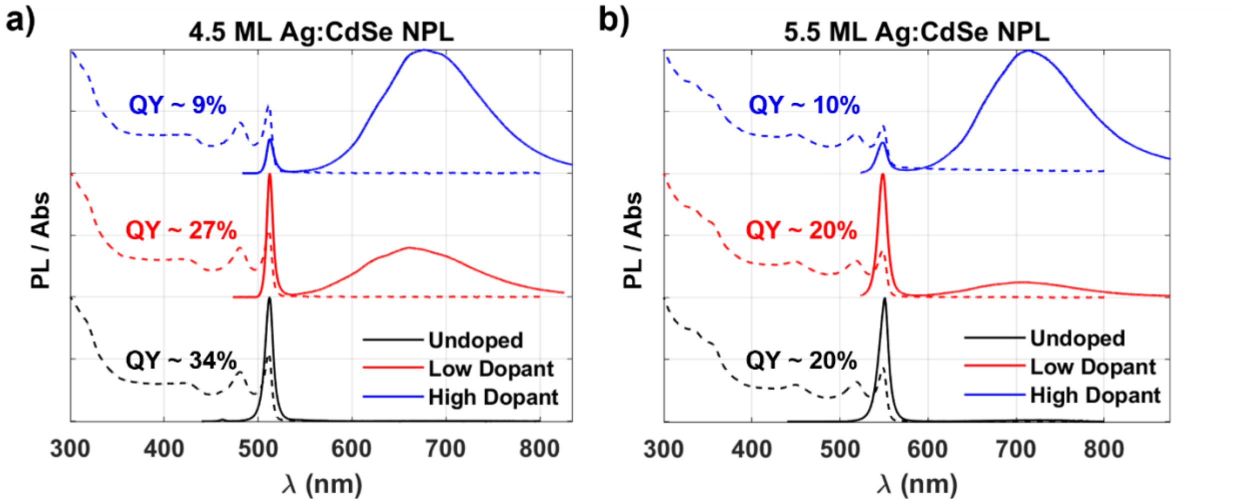

Figure S2. Ensemble characterization of NPL samples (300 K). (a) Solution absorption (dotted) and PL (solid) spectra of 4.5 ML and **(b)** 5.5 ML CdSe NPLs with low and high silver dopant incorporation. The absorption spectra remain unchanged due to Ag-dopants being a mid-gap hole acceptor state. With increasing dopant incorporation, the total PL QY (calculated with a reference dye) decreases from the starting undoped stock solutions – with greater emission intensity arising from the dopant states.

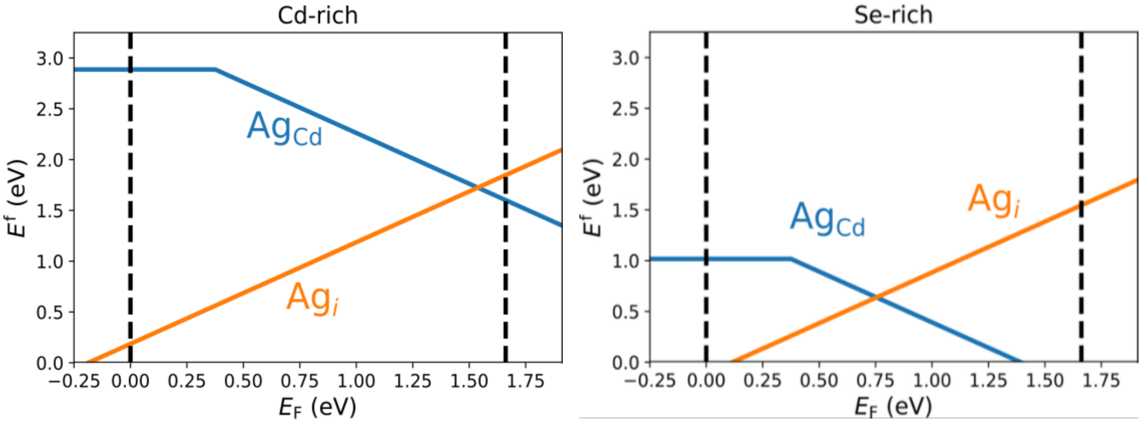

Figure S3. Ag defect formation energies as a function of Fermi level in bulk CdSe. Formation energy of silver on the cadmium site (Ag_{Cd}), which acts as a deep acceptor, is shown in blue. Formation energy of interstitial silver (Ag_i), which acts as a shallow donor, is shown in orange. The band edges are shown by dotted lines, from the valence-band maximum ($E_F = 0$) to the conduction-band minimum. Varying chemical potential conditions, reflecting possible growth conditions, are shown in the two subplots as labeled. Slopes of the lines show the charge state of the defect, so kinks correspond to the thermodynamic charge transition levels. The deep defect level arising from Ag_{Cd} corresponds to its transition from the neutral to the negative state, 0.38 eV above the VBM. The concentration of incorporated silver on each site is proportional to e^{-E^f/k_BT}. These results suggest silver is most likely to incorporate in the acceptor configuration when the nanoplatelets are grown in Se-rich conditions and when the Fermi level during growth is mid-gap or higher.

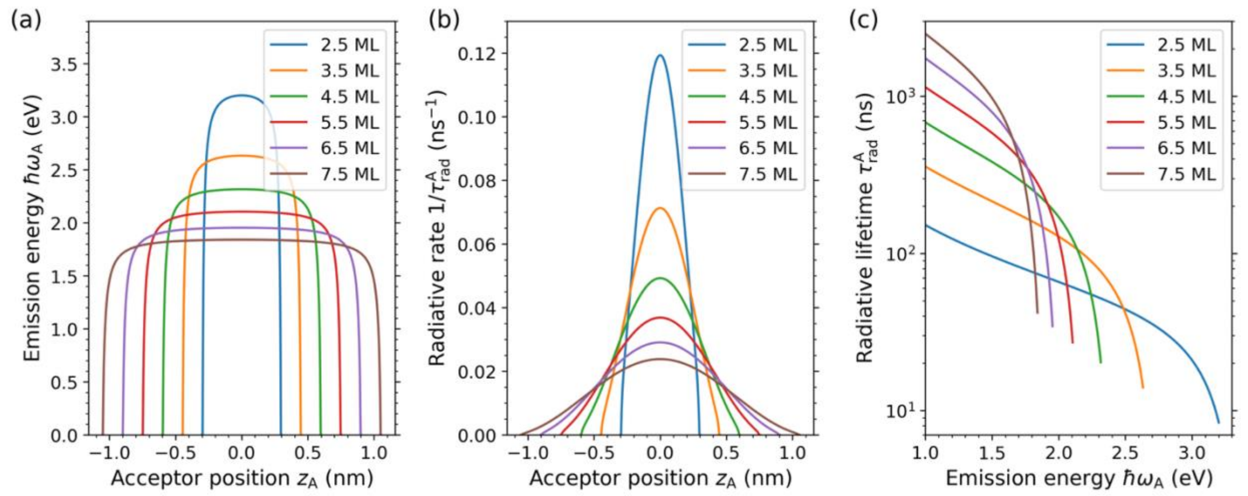

Figure S4. Correlation between emission energy and radiative rate. (a) Emission energy of electron recombination at a silver acceptor as a function of its position z_A, shown for 2.5 through 7.5 monolayer nanoplatelets. Quantum confinement increases the emission energy in thinner nanoplatelets, and self-interaction energy decreases the emission energy for acceptors near the nanoplatelet surface. (b) Radiative rate of the acceptor as a function of z_A. Acceptors near the surface emit more slowly near the surface because of the decreased electron probability density as well as the smaller emission frequency as shown in panel (a). (c) Acceptor radiative lifetime as a function of emission energy.

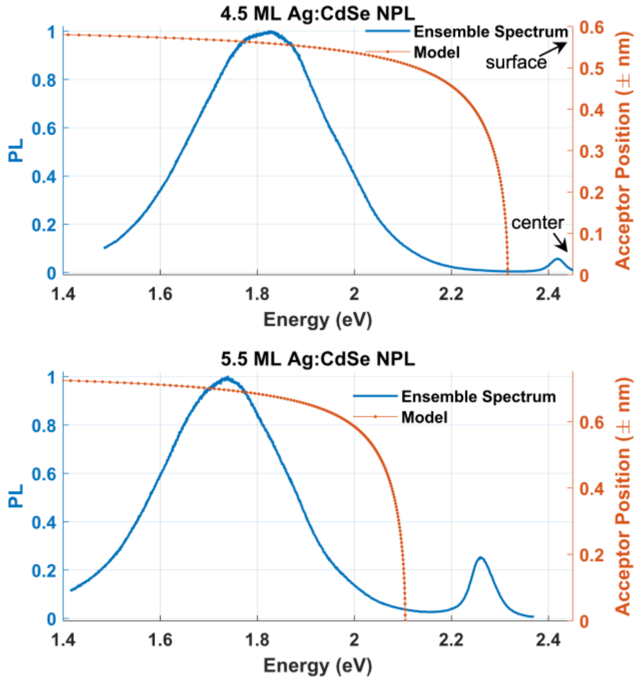

Figure S5. Dopant Emission Energies (Experiment vs. Model). Measured broad dopant emission linewidths are accurately predicted by theoretical model that shows acceptor depth-energy dependence for both 4.5 ML (top) and 5.5 ML (bottom) Ag-doped CdSe nanoplatelets.

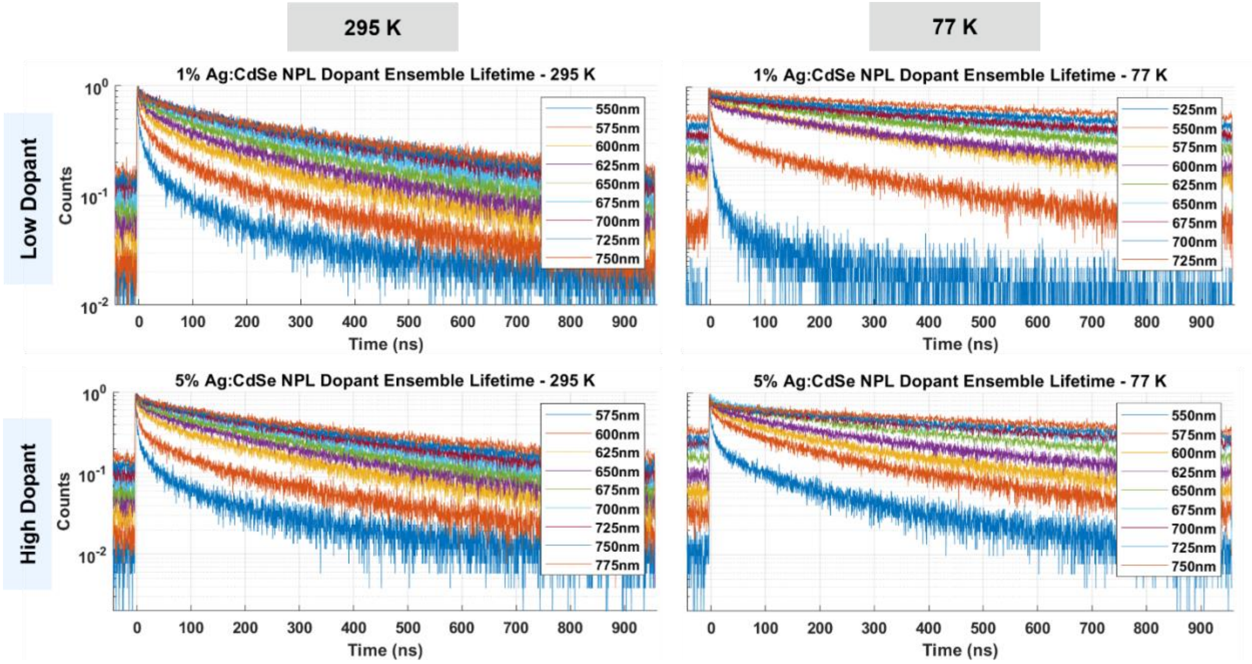

Figure S6. Ensemble 4.5 ML CdSe NPL PL lifetimes vs. dopant emission wavelength. Solution samples are measured in a glass NMR tube with a cold finger dewar for both room temperature and 77 K (liquid-nitrogen) datasets. Dopant PL is filtered using a spectrograph/grating (~3 nm bandwidth) with center wavelength window set every 25 nm. Lifetimes increase for redder dopant emission that correspond to dopant sites located closer to the NPL surface.

Table S2. Individual lifetime component values for low (1%) and high (5%) doped 4.5 ML NPLs as a function of dopant emission wavelength acquired at T= 300 K and T = 77 K.

**4.5 ML Ag:CdSe NPL (1% Ag Doping) T = 300 K Lifetime**							
**λ (nm)**	**A_1**	**t_1 (ns)**	**A_2**	**t_2 (ns)**	**A_3**	**t_3 (ns)**	**τ_{avg} (ns)**
575	187.4	1.81	136.3	20.29	60.48	183.2	37
600	170.8	3.173	140.1	31.44	115.5	229	74
625	200.4	15.6	205.1	226.8			122
650	154.6	20.37	236.7	247.8			158
675	119.3	22.6	276.1	260.1			188
700	97.74	19.33	307	269.5			209
725	87.6	20.93	319.2	281.5			225
750	72.72	20.14	305.7	292			240
775	69.36	16.15	285.8	292			238
**4.5 ML Ag:CdSe NPL (1% Ag Doping) T = 77 K Lifetime**							
525	217	0.8206	150.2	6.018	14.26	116.7	7
550	245.4	2.333	99.89	17.79	104.2	237.9	60
575	118.2	10.13	287	266.9			192
600	152.1	4.18	247	290.9			182
625	54.49	8.34	293.3	320.6			272
650	309.3	322.6					323
675	262.9	342					342
700	250.2	359.3					359
725	233.2	367.9					368

**4.5 ML Ag:CdSe NPL (5% Ag Doping) T = 300 K Lifetime**							
**λ (nm)**	**A_1**	**t_1 (ns)**	**A_2**	**t_2 (ns)**	**A_3**	**t_3 (ns)**	**τ_{avg} (ns)**
575	277.2	2.22	97.69	23.12	42.72	192	27
600	223.5	8.837	126.5	186.9			73
625	184.9	14.7	226.9	221.4			129
650	127.2	17.66	275.7	237.5			168
675	97.16	19.14	306.5	251.9			196
700	83.7	17.15	338.2	264.4			215
725	74.4	14.49	343.8	273.2			227
750	56.9	17.23	343	281.2			244
775	32.56	21.21	333.7	282.5			259
**4.5 ML Ag:CdSe NPL (5% Ag Doping) T = 77 K Lifetime**							
550	174.7	1.013	144.9	14.34	79.64	194.4	44
575	141	2.725	107.5	37.66	178.9	237.9	110
600	155.4	15.49	242.2	243			154
625	122.7	13.84	261.4	274.7			191
650	39.41	16.06	287.9	294.1			261
675	300.2	307.8					308
700	228.4	325.9					326
725	217.8	354.9					355
750	194	364.5					365

Table S3. Individual lifetime component values for low (1%) and high (5%) doped 5.5 ML NPLs as a function of dopant emission wavelength acquired at T= 300 K and T = 77 K.

**5.5 ML Ag:CdSe NPL (1% Ag Doping) T = 300 K Lifetime**							
**λ (nm)**	**A_1**	**t_1 (ns)**	**A_2**	**t_2 (ns)**	**A_3**	**t_3 (ns)**	**τ_{avg} (ns)**
625	247	24.38	129.2	345.7			135
650	219.3	34.21	182.1	413.2			206
675	171.6	47.59	231.3	466.3			288
700	126.8	53.45	278.8	489.4			353
725	86.27	58.23	293.3	521.5			416
750	63.82	52.12	329.3	517.2			442
775	322.7	511.6					512
**5.5 ML Ag:CdSe NPL (1% Ag Doping) T = 77 K Lifetime**							
575	224.2	7.946	94.72	242.6			78
600	151.6	51.18	243.6	393.8			262
625	109.6	68.05	297	462.3			356
650	54.12	50.46	326.9	510.7			445
675	336.2	551.5					552
700	324.9	599.7					600
725	316.1	644.8					645

**5.5 ML Ag:CdSe NPL (5% Ag Doping) T = 300 K Lifetime**							
**λ (nm)**	**A_1**	**t_1 (ns)**	**A_2**	**t_2 (ns)**	**A_3**	**t_3 (ns)**	**τ_{avg} (ns)**
625	217.4	15.67	118.4	293.1			113
650	192.3	28.29	182.2	348.4			184
675	160.8	51.14	217.4	398.1			251
700	148.1	63.3	258.8	424.8			293
725	127.7	77.68	277.2	460.6			340
750	82.35	64.6	308.7	439.7			361
775	84.22	55.28	313.4	459.1			374
800	325.2	454.7					455
**5.5 ML Ag:CdSe NPL (5% Ag Doping) T = 77 K Lifetime**							
575	181.9	8.323	109.2	221.9			88
600	176.7	63.25	225.1	367.2			234
625	131.7	63.35	281	449.5			326
650	90.16	45.83	295.1	511.4			402
675	346.3	492.7					493
700	352.7	521.2					521
725	326	564.1					564
750	292.8	611.5					612

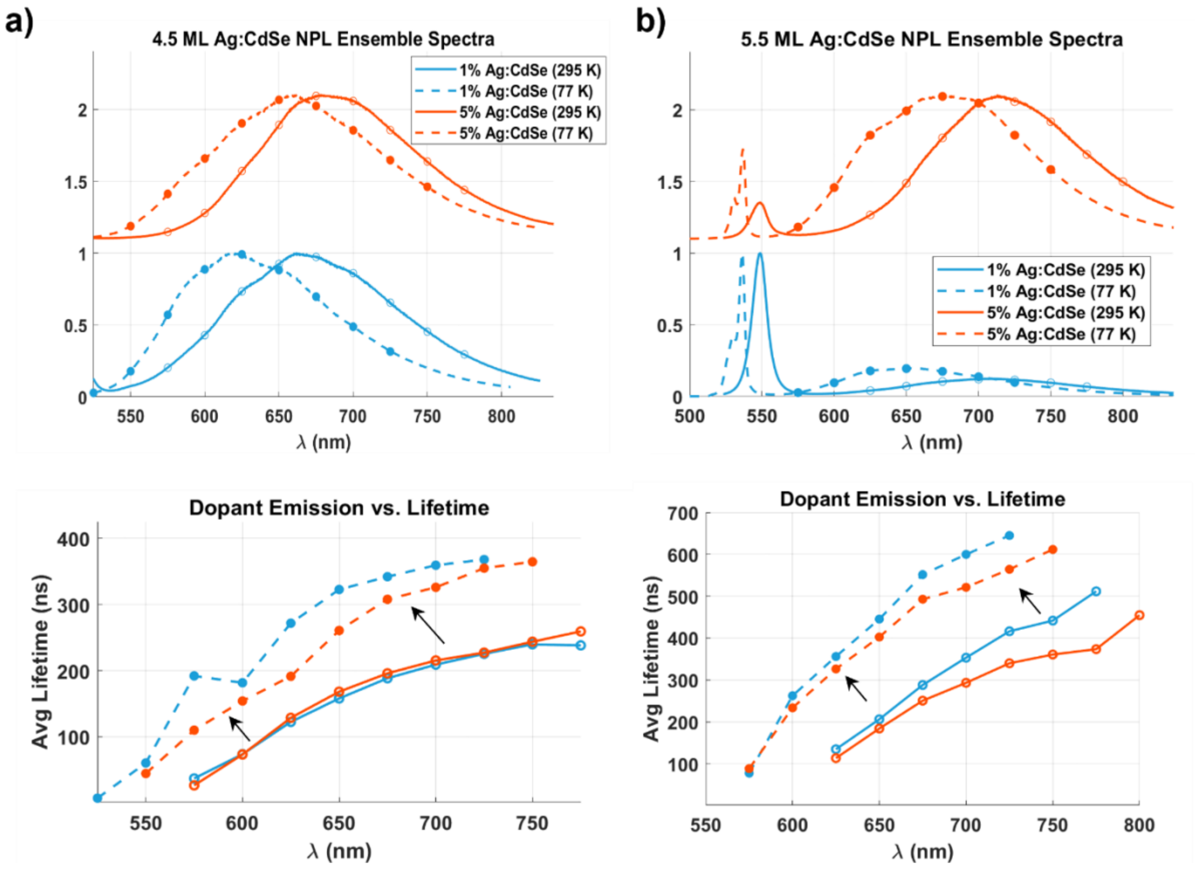

Figure S7. Ensemble 4.5 & 5.5 ML CdSe NPL average PL lifetimes. (a) (top) 4.5 ML NPL low (1%) and high (5%) dopant PL spectra with markers indicating wavelengths at which PL was measured (bottom). Upon cooling, there is an expected blueshift that corresponds to the band-edge exciton temperature dependence. Upon cooling, the dopant linewidth remains largely unchanged and average lifetimes increase. **(b)** 5.5 ML NPLs with similar trend, except in general, lifetimes are longer compared to 4.5 ML.

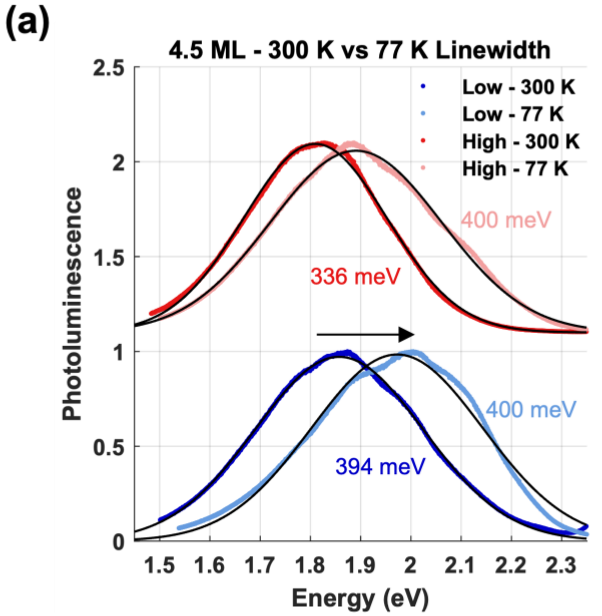

Figure S8. Dopant Emission Linewidth (300 K vs. 77 K). Gaussian fits (black) to dopant emission reveals PL linewidth is largely unchanged upon cooling at low (blue) dopant levels for both (a) 4.5 ML and (b) 5.5 ML NPLs. For high doped samples, we observe a slight increase in the FWHM at 77 K – indicating dopant position is largely responsible for lineshape vs. electron-phonon coupling that is expected to narrow the linewidth at colder temperatures.

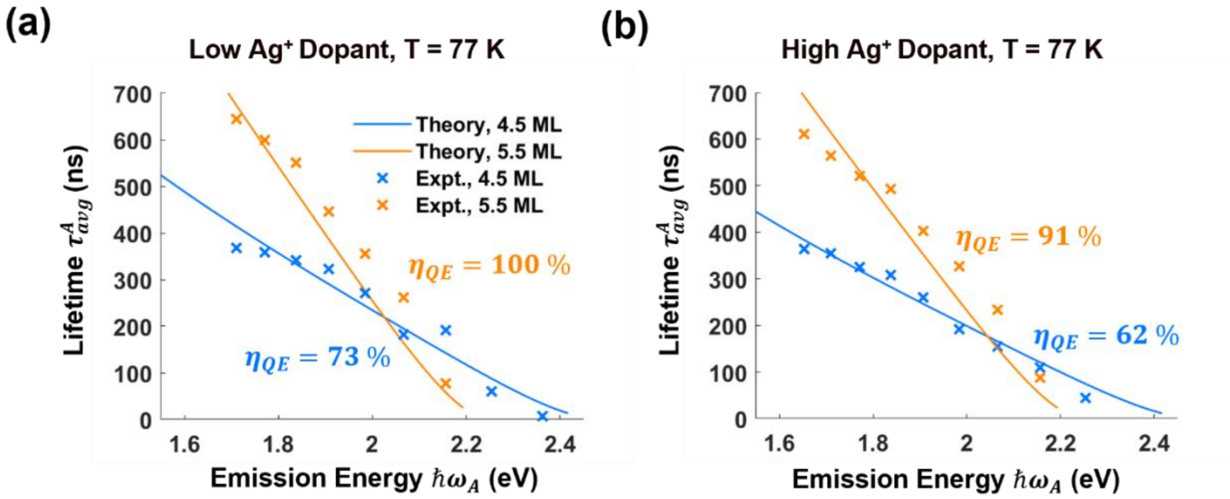

Figure S9. **Lifetime-energy dopant dynamics (77 K). (a)** Corresponding 77 K data for low and **(b)** high dopant incorporation in 4.5 ML & 5.5 ML CdSe NPLs as acquired for room-temperature measurements shown in Fig. 2 (main text). Modeled emissions are rigidly blue-shifted based on the BE blueshift. Our depth-dependent energy-lifetime model is also in good agreement at colder temperatures, indicating spatial dependence of dopant location strongly primarily drives the photophysics. Compared to 300 K, the fitted quantum efficiencies (dopant emission probability once hole is localized on the acceptor) are greater upon cooling, likely corresponding to suppressed non-radiative pathways, as also seen with longer PL lifetimes.

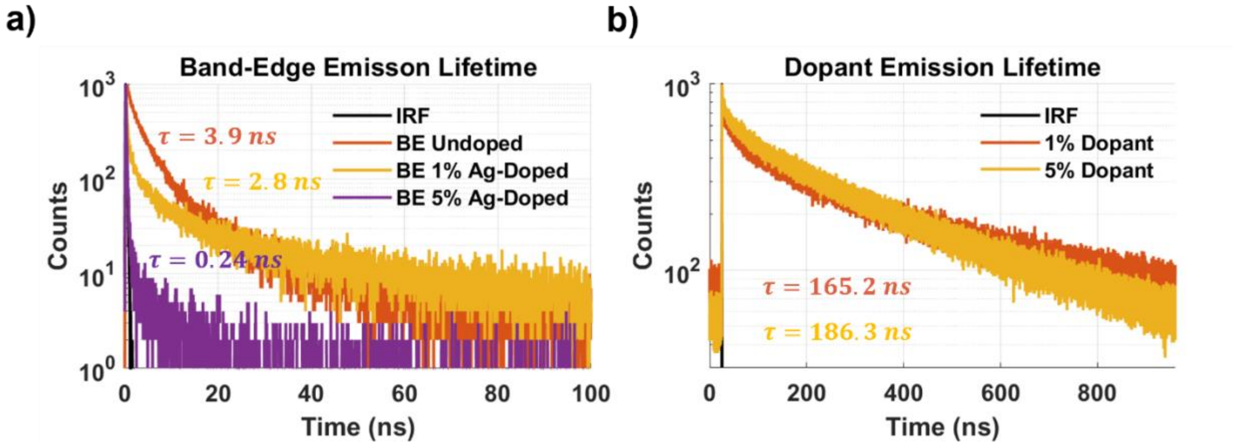

Figure S10. Ensemble 4.5 ML CdSe NPL PL lifetimes (300 K). (a) For the filtered band-edge (BE) only exciton emission, PL lifetimes dramatically reduce upon increasing dopant concentration – strongly indicating quenching due to increasing hole-transfer probability to the silver-acceptor states. **(b)** Filtered dopant emission (across entire dopant spectral window) has nearly 100X longer lifetimes than BE, with higher doped sample having slightly longer lifetime likely due to greater amount of surface dopant sites.

Table S4. Individual time components from exponential tail fitting of the TCSPC curves in Fig. S3. The band-edge (filtered 512 nm) PL quenches and lifetimes shorten significantly with greater Ag-dopant incorporation. The reported amplitude-weighted average lifetime for multi-exponential fitting is determined using:

$$\tau_{avg} = {\sum A_i t_i}/{\sum A_i}$$

4.5 ML Ag:CdSe NPL (Band Edge vs. Dopant) T = 300K Lifetime							
λ (nm)	A_1	t_1 (ns)	A_2	t_2 (ns)	A_3	t_3 (ns)	τ_{avg} (ns)
512 (Undoped)	654.9	1.801	285.9	8.966			3.98
512 (1% Doped)	848.5	0.3174	139.2	5.314	25.01	71.27	2.76
575-775 (1% Doped)	289.1	14.8	427	267.1			165.24
512 (5% Doped)	1024	0.1702	21.55	3.362			0.24
575-775 (5% Doped)	239.3	15.33	533.9	263			186.35

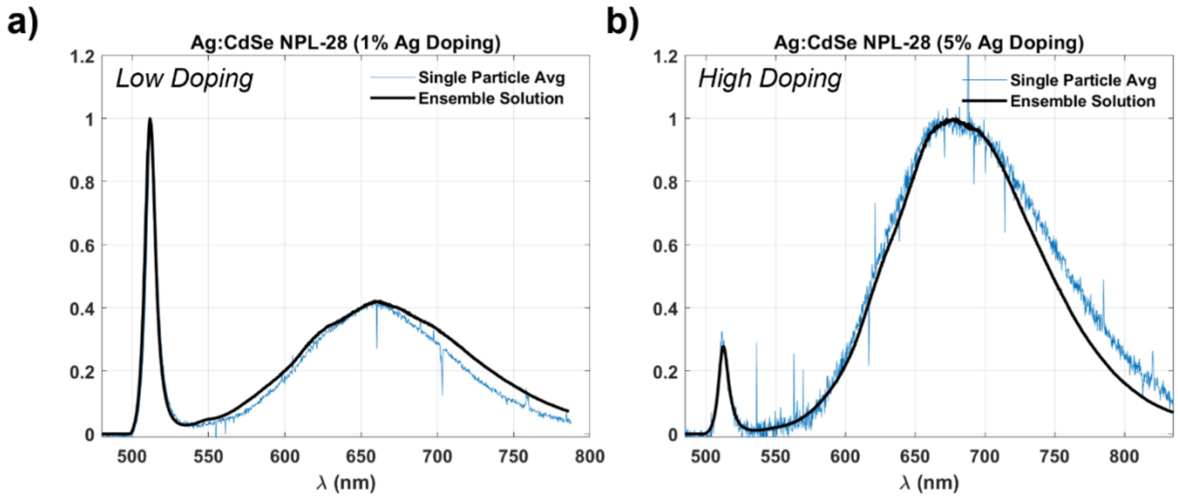

Figure S11. Single particle vs. ensemble spectra. (a) Average of all single particle spectra (50+) shown in Fig. 3 (main text) are in good agreement with ensemble PL spectrum for low and **(b)** high silver-doped samples, confirming the presented single particle statistics are representative of the ensemble photophysics.

Extracting the single photon purity of Ag:CdSe NPLs

The second order photon correlation function was measured in time-tagged mode (TTTR) with the PicoHarp300 time correlator, where the photoluminescence is filtered to include only the broad dopant emission. The raw antibunching curve was fitted as shown in Fig. S8 to the following two-sided mono-exponential function using least-squares fitting in MATLAB:

$$g^2(\tau) = \sum_{i=1}^{11} A_i e^{-|\tau - x_i|/\tau_{LF}} + B$$

where A_i represents the amplitude of the ith peak, x_i is the center location of the corresponding peak, τ_{LF} is the average radiative lifetime of the dopant emission, and B is the background counts accumulated from the combination of dark and substrate scattering over the long 75 min (1 MHz laser excitation) data acquisition per particle. The correlation window span is set to 10 μs, encompassing 11 total peaks (5 on either side of the central zero-time delay (A_6)). The fitting determines the individual peak amplitudes and thus the $g^2(\tau = 0)$ can be extracted by calculating the ratio of the peak area at zero-time difference to average peak areas of the neighboring peaks separated by the laser repetition rate:

$$g^2(0) = A_6\tau_{LF} \Big/ \left(\sum_{i\neq 6} \frac{A_i \tau_{LF}}{10}\right) = A_6 \Big/ \left(\frac{\sum_{i\neq 6} A_i}{10}\right)$$

The single photon emission purity (probability of single photon emission per radiative recombination event) can then be approximated as $1 - g^2(0)$.

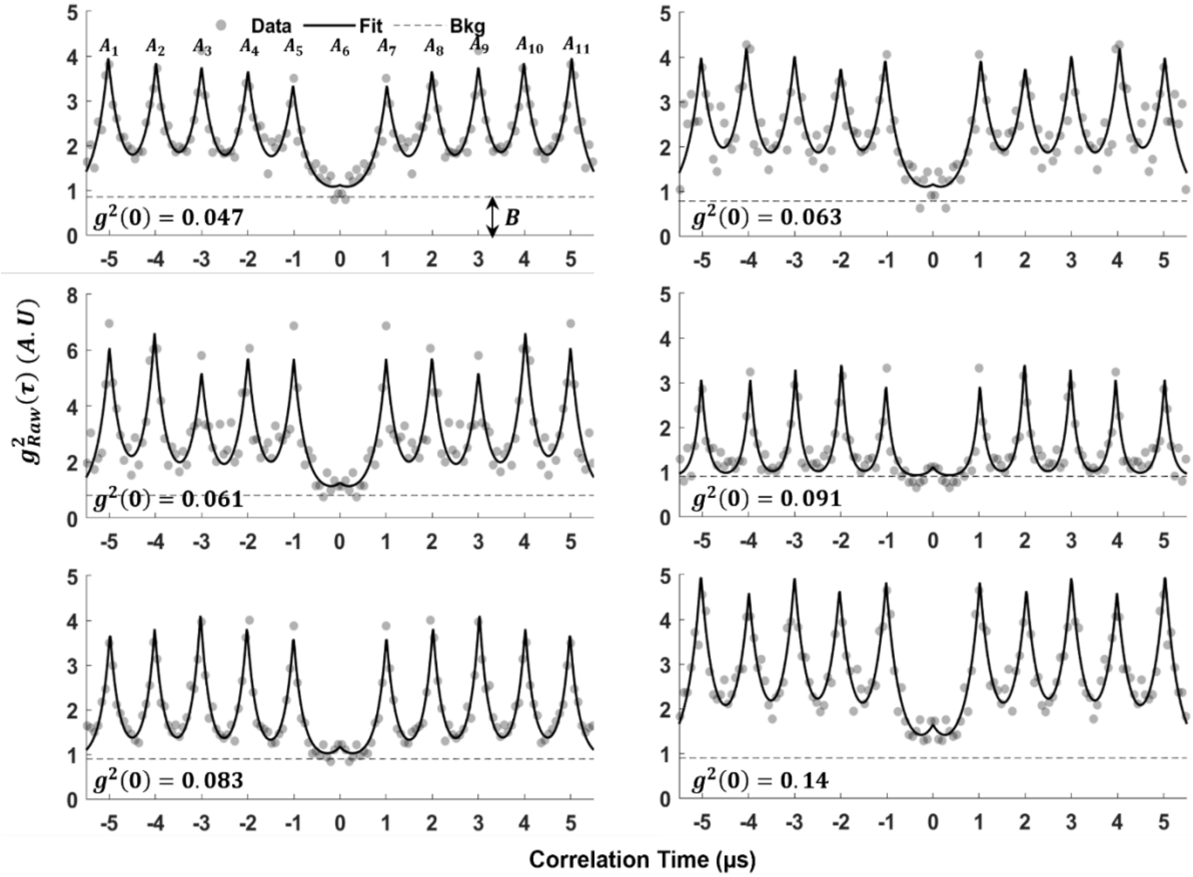

Figure S12. Photon antibunching from low-doped 4.5 ML CdSe NPLs. Second-order (dopant emission state only) intensity correlation functions are measured for 6 example NPL particles with 1 MHz (1000 ns) pulsed excitation with 75 min integration per particle. The data in Fig. 5 (main text) represent averaged antibunching across these 6 particles shown here for an effective integration time of 7.5 hours.

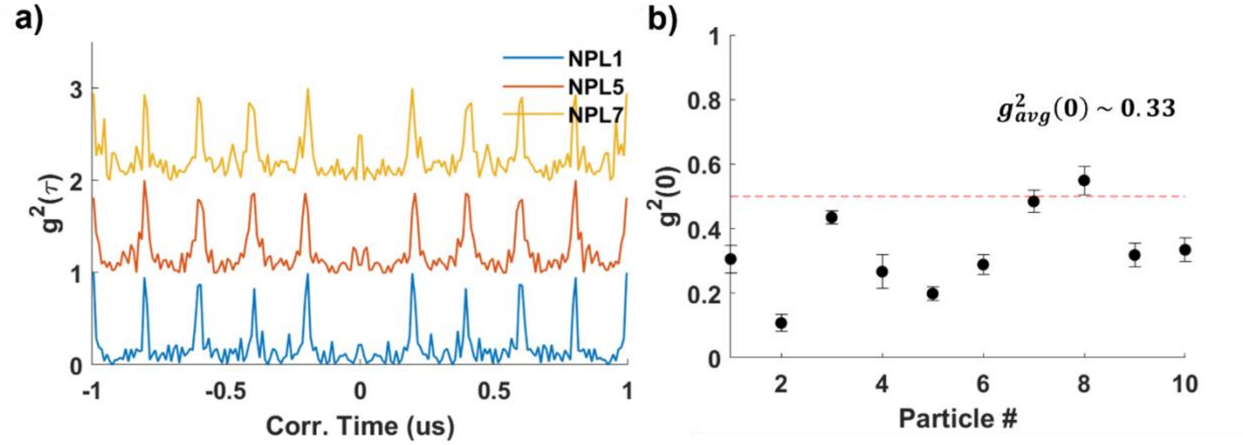

Figure S13. Photon antibunching from undoped 4.5 ML CdSe NPLs. (a) Second-order intensity correlation functions are measured for example NPL particles with 5 MHz (200 ns) pulsed excitation. Suppression of the central t = 0 peak below 0.5 indicates single photon emission. c) Extracted g2(0) values for 10 particles indicated large biexciton QY as high as 50% with some particles showing single photon purity of 70-90 % under ambient conditions.

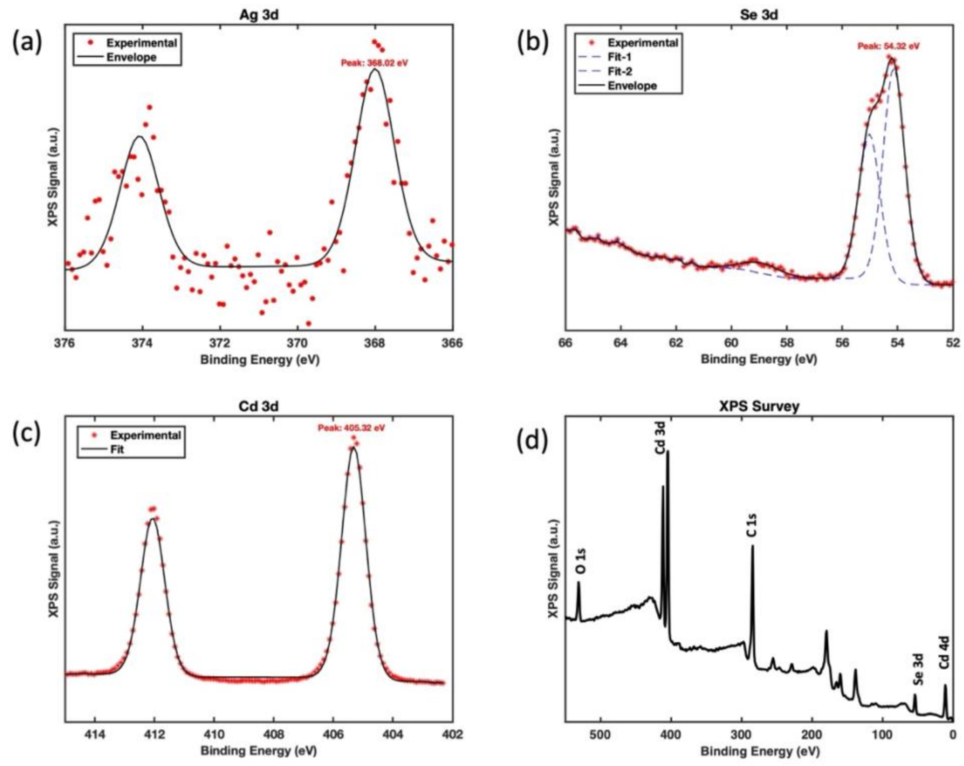

Figure S14. X-ray Photoemission Spectrum from Ag-Doped 4.5 ML CdSe NPLs. (a) Ag 3d, (b) Se 3d, (c) Cd 3d and (d) survey scan.
